# Integrated Four Comparative-Omics Reveals the Mechanism of the Terpenoid Biosynthesis in Two Different Overwintering *Cryptomeria fortunei* Phenotypes

**DOI:** 10.3389/fpls.2021.740755

**Published:** 2021-09-29

**Authors:** Yingting Zhang, Jiebing Cui, Hailiang Hu, Jinyu Xue, Junjie Yang, Jin Xu

**Affiliations:** Key Laboratory of Forest Genetics and Biotechnology of Ministry of Education, Co-innovation Center for Sustainable Forestry in Southern China, College of Forestry, Nanjing Forestry University, Nanjing, China

**Keywords:** cold acclimation, transcriptome, degradome, diterpenoid biosynthesis, metabolome analysis, miRNA target

## Abstract

Chinese cedar (*Cryptomeria fortunei*) is a tree species with important ornamental, medicinal, and economic value. Terpenoids extracted from the essential oil of *C. fortunei* needles have been considered valuable ingredients in the pharmaceutical and cosmetic industries. However, the possible gene regulation mechanisms that limit terpenoid biosynthesis in this genus are poorly understood. Here, we adopted integrated metabolome analysis, transcriptome, small-RNA (sRNA), and degradome sequencing to analyze the differences in terpenoid regulatory mechanisms in two different overwintering *C. fortunei* phenotypes (wild-type and an evergreen mutant). A total of 1447/6219 differentially synthesized metabolites (DSMs)/unigenes (DEGs) were detected through metabolome/transcriptome analyses, and these DSMs/DEGs were significantly enriched in flavonoid and diterpenoid biosynthesis pathways. In *C. fortunei* needles, 587 microRNAs (miRNAs), including 67 differentially expressed miRNAs (DERs), were detected. Among them, 8346 targets of 571 miRNAs were predicted using degradome data, and a 72-miRNA-target regulatory network involved in the metabolism of terpenoids and polyketides was constructed. Forty-one targets were further confirmed to be involved in terpenoid backbone and diterpenoid biosynthesis, and target analyses revealed that two miRNAs (i.e., aly-miR168a-5p and aof-miR396a) may be related to the different phenotypes and to differential regulation of diterpenoid biosynthesis. Overall, these results reveal that *C. fortunei* plants with the evergreen mutation maintain high terpenoid levels in winter through miRNA-target regulation, which provides a valuable resource for essential oil-related bioengineering research.

## Introduction

*Cryptomeria fortunei* (Chinese cedar), an endemic to China, is an evergreen tree species of the family Taxodiaceae. It has become a main garden and timber species in southern China due to its beautiful tree shape, rapid growth and great adaptability, and it is important in ecological environment construction and wood production. Furthermore, it has high value as a traditional medicinal material. Research on the chemical components of *C. fortunei* essential oil is an active field of medicinal research (Lin et al., 2005; Xie et al., 2011). Studies have shown that compounds extracted from *C. fortunei* essential oil have excellent detoxification, antiulcer, antioxidant, antifungal, and insecticidal properties, among others (Matsunaga et al., 2000; Wang et al., 2006; Cheng et al., 2012; Kim et al., 2013); therefore, they have long been used as raw materials in the plant protection, pharmaceutical, and cosmetic industries. Several classes of chemical components, including (oxygenated) mono- (oxygenated), sesqui-, and (oxygenated) diterpenoids, have been shown to be present in the essential oil of *C. fortunei* (Xie et al., 2011). In general, the functional properties of *C. fortunei* essential oils benefit from the presence of a very large number of active ingredients, especially terpenes, terpene alcohols, and flavonoids (Cheng et al., 2009). According to previous reports, within *Cryptomeria*, there are certain differences in the chemical composition of its essential oils among different species, trees of different ages and different places of production, and different tree organs or tissues (Lin et al., 2005; Cheng et al., 2009; Xie et al., 2011). Therefore, it is necessary to understand the mechanism underlying terpenoid synthesis in *C. fortunei* and to enhance its production overall.

Among the plant specialized metabolites, terpenoids are the most diverse and abundant; to date, more than 75,000 terpenoids have been discovered (Fujihashi et al., 2018). Although the structures of plant terpenoids vary greatly, the synthetic precursors of all plant terpenoids are the five-carbon (C5) compound isopentenyl diphosphate (IPP) and its isomer dimethylallyl diphosphate (DMAPP), which undergo rearrangement and cyclization reactions resulting in the synthesis of various terpenoid skeletons. Terpenoids can be divided into hemi- (C5), mono- (C10), sesqui- (C15), di- (C20), tri- (C30), tetra- (C40), and polyterpenoids (C > 40) (Ashour et al., 2010) based on the number of C5 units contained in the molecule. Studies have shown that terpenoid synthesis involves two biosynthetic pathways: the mevalonic acid (MVA) pathway, through which sesqui- and triterpenoids are produced, and the 1-deoxy-D-xylosyl-5-phosphate (DOXP)/2-C-methyl-D-erythritol-4-phosphate (MEP) pathway, through which monoterpenoids, diterpenoids, tetraterpenoids, zeatin, and other terpenoid quinones are synthesized (Vranová et al., 2013). In the life activities of plants, terpenoids are important in signal transduction, resistance to (a)biotic stress, and biological interactions (Izumi and Hirata, 1997; Grassmann et al., 2002).

MicroRNAs (miRNAs) are endogenous non-coding RNAs with a length of 21–25 nt and play a vital regulatory role in plant growth and development and adaptation to environmental changes. Specifically, they are important in flower transformation and development, senescence, and (a)biotic stress responses (Lu and Huang, 2008; Song et al., 2013; Xu et al., 2013). In recent years, as high-throughput technology has developed, small-RNA (sRNA) sequencing has been widely used in the identification of plant miRNAs, including those of the camphor tree (*Cinnamomum camphora*) (Chen et al., 2019), Masson pine (*Pinus massoniana*) (Ye et al., 2020), and ginkgo (*Ginkgo biloba*) (Jia et al., 2020). In addition, miRNAs play a very important role in regulating gene expression through their ability to shear target messenger RNAs (mRNAs) and to inhibit the translation of targeted mRNAs (Bartel, 2004). Computer algorithms such as TargetScan (Grimson et al., 2007), MiRanda (Doron et al., 2008), and PicTar (Krek et al., 2005) are the methods most commonly used to identify miRNA targets. However, the miRNA targets that are currently known are very limited and do not provide a sufficient basis for prediction, and the identification of predicted candidate genes is relatively cumbersome; therefore, it is difficult to achieve high-throughput and large-scale identification. With the emergence of degradome sequencing (degradome-seq), high-throughput identification of plant miRNA target genes has become possible; therefore, this method has been widely used to predict plant miRNA target genes. For example, *C. camphora* (Chen et al., 2019), *P. massoniana* (Ye et al., 2020), and kidney bean (*Phaseolus vulgaris*) (Parreira et al., 2021) have all been shown to harbor a variety of miRNA targets. In recent years, the role of miRNAs and their targets in regulating the biosynthesis and accumulation of specialized metabolites such as flavonoids, terpenoids, and alkaloids in various plants has been reported. For example, in *Arabidopsis thaliana*, miR156 targets *squamosa promoter binding protein-like* (*SPL9*) and negatively regulates (E)-β-caryophyllene biosynthesis (Yu et al., 2015), and in bitter gourd (*Picrorhiza kurroa*), miR4995 targets *3-deoxy-7-phosphoheptulonate synthase* (*DHS*) and affects the production of picroside-I by regulating terpenoid biosynthesis (Vashisht et al., 2015). However, there are few reports concerning the regulation of terpene synthesis by miRNAs and their transcription factors (TFs)/target genes in *C. fortunei*; therefore, further research is needed to better understand the regulatory role of miRNAs in the production of terpenoids.

In this study, using two phenotypes of *C. fortunei* needles [normal (wild-type with yellowish-brown needles in winter, YWt) and an evergreen mutant (GM)] in winter as materials, transcriptome, and metabolome analyses were used to identify differentially synthesized metabolites (DSMs) and unigenes (DEGs), respectively. These DSMs/DEGs were significantly enriched in terpenoid-related pathways. Then, through analysis of the sRNAs and the degradome, miRNAs present in *C. fortunei* needles and their targets were further identified, and the role of the miRNA-target module in terpenoid biosynthesis was analyzed. Our results reveal the presence of differential molecular mechanisms of terpenoid biosynthesis in different *C. fortunei* phenotypes in winter; this finding helps direct the selection of *C. fortunei* for essential oil extraction and provides a theoretical basis for its further genetic improvement to increase its terpenoid content in winter.

## Materials and Methods

### Plant Material and Extraction of Total RNA

Two representative *C. fortunei* clones with different overwintering phenotypes [#X1 (GM) and #3 (YWt)] cultivated in the Garden Experimental Teaching Center of Nanjing Forestry University (32°04’41″N, 118°48’43″E, Nanjing, Jiangsu, China) were selected as experimental materials. For each clone, needle samples were collected from among the upper and middle needles of the secondary lateral branches of 5-year-old trees. According to our previous research and related references, February is the period in which *Cryptomeria* shows the greatest phenotypic differences (Han et al., 2003). Therefore, the needle samples were collected on February 15, 2020, and all samples were immediately stored at −80°C. Three biological replicates were used as materials for transcriptomic analysis and sRNA sequencing, and six biological replicates were used for metabolome analysis (quantitative analysis of all metabolites).

Total RNA of *C. fortunei* was extracted using the RNeasy Plant Mini Kit (Qiagen, Hilden, North Rhine-Westphalia, Germany) following the manufacturer’s instructions. RNA integrity was evaluated with an Agilent 2100 Bioanalyzer (Agilent Technologies, Santa Clara, CA, United States), and only RNA with an RNA integrity number (RIN) value >7.0 was used for high-quality sequencing.

### *De novo* Transcriptome Sequencing

Transcriptome sequencing was conducted by OE Biotech Co., Ltd. (Shanghai, China). To obtain clean reads (CRs), adaptor sequences, low-quality reads, and poly-N sequences were removed from the raw data using Trimmomatic version 0.36 (Bolger et al., 2014) with the following parameters: LEADING: 3, TRAILING: 3, SLIDINGWINDOW: 4:15, and MINLEN: 50. The CRs were then assembled *de novo* into transcripts using Trinity version 2.4.0 (Grabherr et al., 2013) in paired-end mode, with RF and SS_lib_type as the parameters. The longest transcript was selected as a unigene according to the sequence length and similarity and used in the subsequent functional annotation and expression calculation.

The unigenes were annotated through alignment with the National Center for Biotechnology Information (NCBI)’s non-redundant (Nr), eukaryotic orthologous groups of proteins (KOG), and SwissProt databases using blastx (Altschul et al., 1990) with a threshold *E*-value of 10^–5^. These resulting SwissProt IDs were mapped to the Gene Ontology (GO) database, and GO annotations of the unigenes were obtained. Finally, the unigenes were compared to the Kyoto Encyclopedia of Genes and Genomes (KEGG) database (Kanehisa et al., 2008) to obtain pathway information.

The fragments per kilobase of exon model per million mapped fragments (FPKM) values (Trapnell et al., 2010) of each unigene were calculated using bowtie2 version 2.3.3.1 (Langmead and Salzberg, 2012). The functions estimateSizeFactors and nbinomTest in DESeq version 1.26.0 (Anders and Huber, 2016) were used to identify DEGs, and *p*-values < 0.05 and fold change >2 or <0.5 were set as their thresholds. KEGG and GO analyses of these DEGs were performed using R according to the hypergeometric distribution to predict the biological functions and pathways mainly affected by the DEGs. Hierarchical cluster analysis of these DEGs was performed to explore the expression patterns of the related transcripts.^1^

### sRNA Library Construction and Sequencing

High-quality CRs were obtained after removing adaptor sequences, sequences <15 or >41 bp in length, reads containing N bases, and low-quality sequences from the raw reads (RRs). The length distribution of CRs was determined to preliminarily evaluate the sRNA distribution of the sample. The CRs were compared to the transcriptome to determine the comparison rate. Non-coding RNAs were annotated as ribosomal RNAs (rRNAs), transfer RNAs (tRNAs), small nuclear RNAs (snRNAs), and small nucleolar RNAs (snoRNAs). The RNAs were aligned and then subjected to a blastn search (Altschul et al., 1990) against the Rfam version 10.0^2^ and GenBank databases.^3^

Known miRNAs were identified through alignment with the miRBase database version 21.0.^4^ Unannotated sRNAs were analyzed using miRDeep2 version 2.0.0.8 (Friedlnder et al., 2012) to predict novel miRNAs. The miRNA expression levels were quantified as transcripts per kilobase million (TPM) values. DESeq version 1.26.0 (Anders and Huber, 2016) was used to standardize the expression of miRNA in each sample and to calculate the fold change, and the negative binomial distribution test method was used to test the significance of the difference in miRNA expression data. Differentially expressed miRNAs (DERs) were identified with the threshold of *p*-value < 0.05 and | log2FoldChange| > 1.

### Degradome-seq and Target Gene Identification

To construct degradation libraries, two 30-μg aliquots of total RNA extracted from the GM and YWt phenotypes, respectively, of *C. fortunei* were purified using the Total RNA Purification Kit (LC Science, Houston, TX, United States). The mRNAs were connected to 3′ and 5′ adaptors, reverse-transcribed using a mixture of biotinylated random primers and mRNAs, and amplified by polymerase chain reaction (PCR) to complete library construction. These constructed libraries were sequenced using an Illumina HiSeq 2500 sequencer (Illumina, San Diego, CA, United States). The single-end read length was 1 × 50 bp, and the sequencing data contained more than 5 million reads.

The raw sequencing data were filtered using Illumina Pipeline version 1.5. The miRNA targets and their cleavage sites were predicted using the CleaveLand program version 4.0 (Addo-Quaye et al., 2009) and mapped to the *C. fortunei* transcriptome data. Based on the abundance and characteristics of superaccumulated single genes in the *C. fortunei* transcriptome data, a degradome density file was established. According to the abundance of cleavage site reads (CSRs), miRNA targets can be divided into five categories as the reliability decreases: category 0 (1 maximum CSR), category 1 (>1 maximum CSR), category 2 (CSR abundance > median transcript abundance), category 3 (CSR abundance ≤ recorded median transcript abundance), and category 4 (1 RR containing the cleavage site). These target mRNA sequences that matched an sRNA sequence from the sequenced species were predicted by TargetFinder software (Lavorgna et al., 1999) with default parameters. To further study miRNAs and their targets, KEGG and GO analyses were performed on all targets.^5^ Subsequently, these interaction relationships were applied to construct the entire miRNA–gene interaction network, and the network was then visualized in Cytoscape version 3.5.1.^6^

### Metabolomics Study

An accurately weighed 80-mg needle sample was transferred to a 1.5-mL microcentrifuge tube and extracted using 20 μL of 1.5 mM 2-chloro-L-phenylalanine and 1 mL of 70% methanol. The precooled sample was ground at 60 Hz for 2 min, sonicated at ambient temperature for 0.5 h, allowed to stand at −20°C for 20 min, and centrifuged at 13,000 rpm at 4°C for 10 min. Then, 300 μL of the supernatant was evaporated to dryness and reconstituted with 400 μL of 20% methanol. The sample was vortexed well, precooled at 4°C for 2 min and centrifuged for 10 min. Finally, 150 μL of the supernatant was aspirated using a syringe, filtered, and transferred to a liquid chromatography (LC) injection bottle, and stored at −80°C for liquid chromatography–mass spectrometry (LC–MS) determination.

An LC–MS system consisting of an AB ExionLC ultra-high-performance LC instrument in tandem with an AB TripleTOF 6600 plus high-resolution mass spectrometer was used to analyze the metabolic spectrum in positive and negative ESI modes. An ACQUITY UPLC HSS T3 (100 mm × 2.1 mm, 1.8 μm) column was used, and water containing 0.1% formic acid and acetonitrile (*v*/*v*) was used as a binary mobile phase for gradient separation. Sample MS signal acquisition was performed in two ESI modes, and quality control (QC) samples were introduced throughout the entire analysis process to evaluate repeatability. The raw data collected by UNIFI 1.8.1 were processed by the metabolomics processing program Progenesis QI version 2.3 (Non-linear Dynamics, Newcastle, United Kingdom), and identification was then performed based on http://www.hmdb.ca/, http://www.lipidmaps.org/ and a self-built database. For the extracted data, the resulting matrix was further reduced through removing peaks for which values were missing in >50% of the samples. Finally, both ion mode data were merged into a table that was imported into the R ropls package.

After mean centring and Pareto variance scaling, principal component analysis (PCA) and (orthogonal) partial least squares-discriminant analysis [(O)PLS-DA] were performed to visualize the metabolic differences between the experimental groups. In the model score graph, the T2 area of Hotelling shown in the oval shape defines the 95% confidence interval of the model variables. The variable importance in the projection (VIP) ranks the overall contribution of each variable to the OPLS-DA model; VIP >1 indicates a potential biomarker. The default sevenfold cross-validation and 200-response permutation testing methods were used to examine the quality of the model to prevent overfitting. In addition, DSMs were selected based on a combination of the statistical significance threshold of the VIP value obtained from the OPLS-DA model and the *p*-value of the normalized peak area of the two-tailed Student’s *t*-test. Metabolites with VIP values >1.0 and *p*-values < 0.05 were considered DSMs. In addition, DSMs were mapped to the KEGG database to obtain their common pathway information. The KEGG IDs of the DSMs were used to perform enrichment analysis to obtain the enrichment results of the metabolic pathways. A hypergeometric test was then applied to find pathway entries that were significantly enriched in DSMs compared with the entire background metabolites. The bubble chart of KEGG pathway enrichment analysis was visualized based on https://cloud.oebiotech.cn/task/.

### Validation of DERs

Nine DERs were selected for determination of expression levels using quantitative real-time PCR (qRT-PCR). Based on these obtained sequences, gene-specific primers were designed (Supplementary Table 1).

MicroRNAs were extracted from 0.1 g needle samples using the miRNA Isolation Kit (Tiangen Biotech Co., Nanjing, Jiangsu, China) following the manufacturer’s instructions. Qualified samples that had been evaluated for integrity, purity, and concentration were used to synthesize first-strand cDNAs using the miRcute Plus miRNA First-Strand cDNA Synthesis Kit (Tiangen Biotech Co.). qRT-PCR was performed on the same instrument as that used by Zhang et al. (2021) with the following amplification procedure: 95°C predenaturation for 15 min followed by 40 cycles of template denaturation and annealing; a dissociation curve was generated at 60–95°C. The 2^–Δ^ ^Δ^ ^Ct^ method was used to calculate the relative expression levels with *U6* and cln-miR6725 as references (Supplementary Table 1). All experiments were performed in triplicate, and each sample was examined in three technical replicates.

## Results

### Metabolomic Profiling Analysis

**FIGURE 1:**
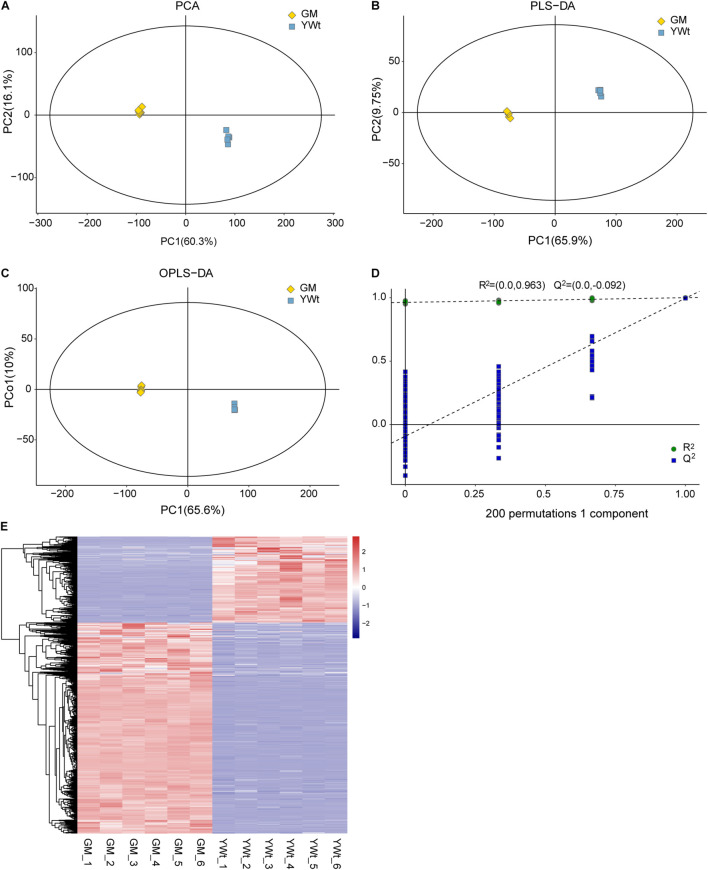
Multivariate statistical analysis of the metabolome and differentially synthesized metabolites (DSMs). Plots of principal component analysis (PCA) **(A)**, partial least squares discriminant analysis (PLS-DA) **(B)**, and orthogonal PLS-DA (OPLS-DA) **(C)**. **(A,B)** The *x*- and *y*-axes represent the first (PC1) and the second (PC2) principal components, respectively; **(C)** the *x*- and *y*-axes represent predictive principal components and orthogonal principal components, respectively. **(D)** The 200-response sorting tests of the OPLS-DA model. **(E)** Hierarchical clustering heatmap of DSMs. Each column and row represent a sample and a differentially expressed unigene (DEG), respectively, and the colors indicate the expression levels of DSMs. GM, evergreen mutant; YWt, yellowish-brown needles in winter; GM_1, GM_2, GM_3, GM_4, GM_5, and GM_6 represent six repetitions.

To investigate the metabolic differences between the *C. fortunei* phenotypes, we analyzed the non-targeted metabonome of *C. fortunei* needles using LC–MS. The total ion current of 12 samples showed large peak capacity and excellent retention time reproducibility (Supplementary Figure 1), indicating that the difference between biological replicates and the error caused by the instrument were both small. In these needles, a total of 9251 compounds with definite formulas were identified (MTBLS3183), and the parameters for metabolite annotation were described according to the guideline by Fernie et al. (2011), including 5815 lipid-like molecules, accounting for 62.86% of all metabolites. Among them, 173 metabolites were identified as terpenoids, including 87 sesqui-, 35 di-, 32 mono-, 18 tri-, and 1 polyterpenoid(s) (Supplementary Table 2). PCA and (O)PLS-DA accurately divided all the samples into two different clusters, reflecting the obvious difference between green and yellow needles (Figures 1A–C). The accuracy of the OPLS-DA model was also tested using sevenfold cross-validation (Figure 1D).

**FIGURE 2:**
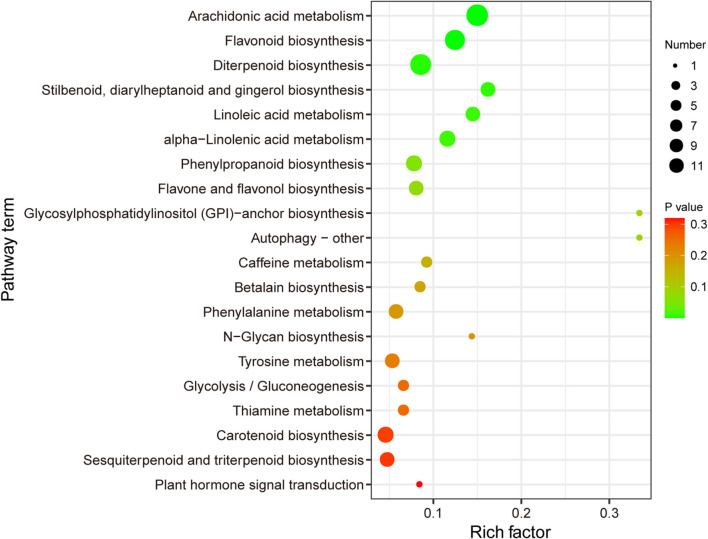
Kyoto Encyclopedia of Genes and Genomes analysis of differentially synthesized metabolites (DSMs). The *x*- and *y*-axes represent the enrichment factor and the pathway term, respectively. The colors and sizes of the dots represent the significance (*p*) and the number of metabolites, respectively.

A total of 1447 DSMs were detected by combining single- and multidimensional analysis; these DSMs included 31 terpenoids (10 di-, 6 mono-, and 15 sesquiterpenoids) (Supplementary Table 3), and the synthesis of most metabolites was enhanced in GM (Figure 1E and Supplementary Table 4). In addition, 111 DSMs were enriched in 56 metabolic pathways, of which the top three were “arachidonic acid metabolism” (ath00590), “flavonoid biosynthesis” (ko00941), and “diterpenoid biosynthesis” (ko00904) (Figure 2). Therefore, we preliminarily speculated that *C. fortunei* that display different phenotypes may differentially accumulate these metabolites.

### Assembly, Annotation, and Enrichment Analysis of the *C. fortunei* Transcriptome

To identify genes expressed in *C. fortunei* needles, six transcriptome libraries (GM_1, GM_2, GM_3, YWt_1, YWt_2, and YWt_3) were generated using total RNA extracted from three biological replicates of each phenotype. A total of 304.79 M RRs were obtained, and the sequencing results were submitted to the NCBI database under registration number PRJNA697258 (SAMN17672908–17672913). The low-quality sequences were filtered out, and in total, 302.75 M CRs were obtained; the Q30 (%) was >95.57%, and the gas chromatography (GC) content was 44.19–44.33% (Supplementary Table 5). Then, 55,839 single unigenes (submission number: SUB10083705, Supplementary Tables 6, 7) were assembled using Trinity software. The N50 value and the average length were 1219.86 and 1990 bp, respectively. The unigenes expressed in the GM and YWt samples clearly aggregated into different clusters, indicating that the biological reproducibility was good and that the samples could be used for subsequent analysis (Supplementary Figure 2). Finally, through BLAST comparison with seven public databases, a total of 27,769 single unigenes were annotated, of which 27,144 unigenes that significantly matched the Nr database accounted for the highest percentage of the total (48.61%) (Supplementary Tables 6, 8).

**FIGURE 3:**
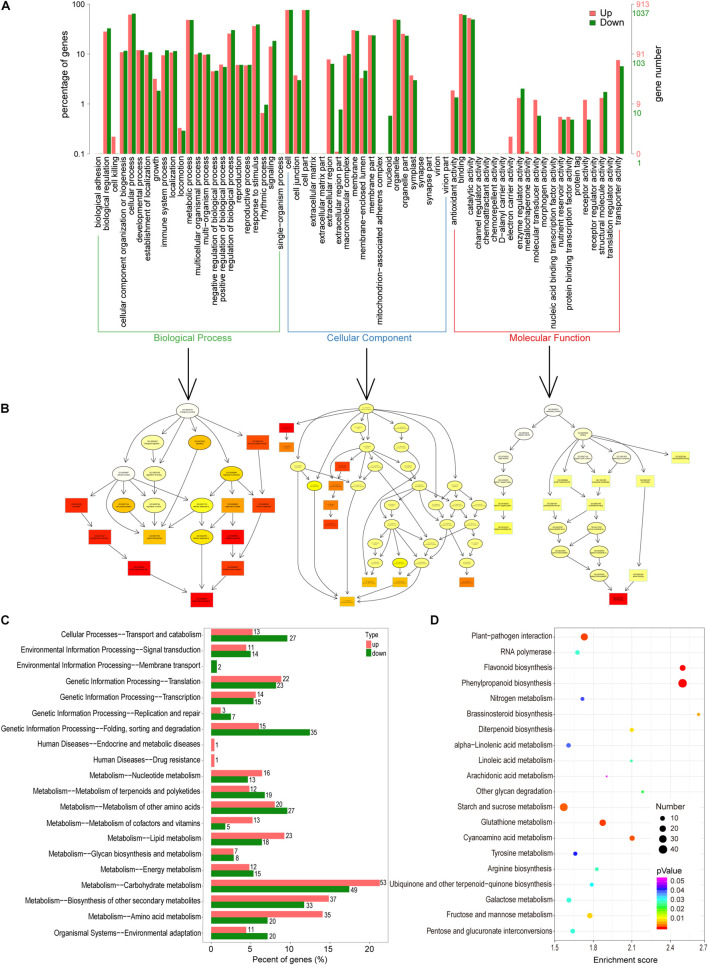
Functional classification of differentially expressed unigenes (DEGs). **(A)** Comparison of the distribution of DEGs at the GO level. Red/green indicates GO entries enriched for up-/downregulated DEGs; the *x*-axis is the name of the entry, and the *y*-axis represents the number of unigenes in the corresponding entry and its percentage. **(B)** Directed acyclic graphs of three main categories. The 10 most significant nodes are represented by rectangles; the colors of the rectangles represent the significance of enrichment, and the significance increases as the color changes from yellow to red. The basic information on each node is displayed in the corresponding graph. **(C)** KEGG distribution map of DEGs. The *x*-axis shows the up- and downregulated DEGs annotated to each pathway and all these DEGs annotated to the KEGG pathway to the total number (%); the *y*-axis indicates the name of the pathway, and the number on the right side of the column indicates the number of DEGs annotated to each pathway. **(D)** KEGG enrichment analysis of DEGs. The *x*- and *y*-axes represent the enrichment score and the pathway term, respectively. The colors and sizes of the dots represent the significance (*p*) and the number of unigenes, respectively.

In total, 6219 DEGs were detected when the GM and YWt libraries were compared; these included 3096 upregulated and 3123 downregulated DEGs (Supplementary Figure 3). Classification of these DEGs by GO function yielded 1950 DEGs, including 913 upregulated and 1037 downregulated DEGs. The enrichment histogram showed that the most enriched category was “biological process” (BP), followed by “cellular component” (CC) and “molecular function” (MF) (Figure 3A). In BP, the most enriched subcategory was “biological regulation,” followed by “metabolic process” and “response to stimulus”; “cell,” “cell part,” and “organelle”; and “binding,” “catalytic activity,” and “transporter activity” were the three most enriched subcategories in CC and MF, respectively. Directed acyclic graphs were used to display the results of the GO structure, and the GO terms were enriched in the following order: CC, MF, and BP (Figure 3B). A total of 669 DEGs were enriched in KEGG pathways; the most significantly enriched pathway was “carbohydrate metabolism,” followed by “biosynthesis of other specialized metabolites” (Figure 3C); 805 DEGs were enriched for 112 KEGG terms, and the most highly enriched ones were “phenylpropanoid and flavonoid biosynthesis,” “glutathione metabolism,” “plant-pathogen interaction,” “cyanoamino acid metabolism,” “starch and sucrose metabolism,” “brassinosteroid biosynthesis,” “fructose and mannose metabolism,” and “diterpenoid biosynthesis” (Figure 3D).

### High-Throughput sRNA Sequencing

To identify the DERs related to the phenotypic differences between the two *C. fortunei* strains, six independent sRNA libraries were generated in February, and the sequencing results were submitted to the NCBI database; the sequence read file registration number is PRJNA720228 (SAMN18644350–18644352, 18644359–18644361). A total of 254.59 M RRs were obtained, and 21.00–26.99 M CRs were obtained in each library after removal of low-quality reads, poly-N sequences, adaptor sequences, and reads <15 or >41 bp in length (Supplementary Table 9). A total of 120,677,469 reads were mapped to the aforementioned transcriptome; approximately 0.146% (208,203) of the sRNA reads were mapped to non-coding RNAs (i.e., rRNAs, tRNAs, snRNAs, and *cis*-regulatory elements) in the Pfam database, and an average of 465,479.33 localization reads in each library were recognized as known miRNAs (Supplementary Table 9). The most abundant sequence among the *C. fortunei* sRNAs was the 21-nt sRNA tag (Supplementary Figure 4).

**FIGURE 4:**
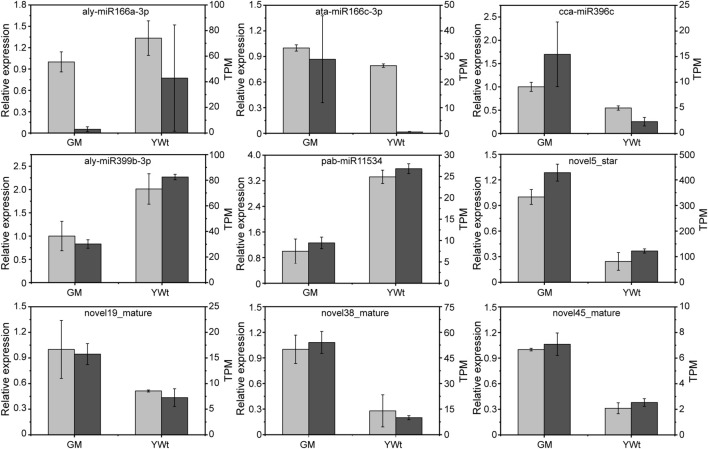
Quantitative real-time PCR (qRT-PCR) validation of selected DERs. The *x*- and *y*-axes represent the sample (GM, evergreen mutant; YWt, yellowish-brown needles in winter) and the transcripts per kilobase million (TPM) or relative expression level, and the values shown represent the mean ± SD; *n* = 3.

A total of 587 miRNAs were detected; 439 were expressed in both phenotypes, and 67 and 81 were expressed in only GM or YWt, respectively (Supplementary Figure 5A). A total of 391 miRNAs were identified as known miRNAs, 359 of which were distributed in 40 miRNA families (Supplementary Figure 5B). Sixty-seven DERs showed significant differences between the two phenotypes, including 29 downregulated and 38 upregulated miRNAs in GM (Supplementary Figure 6). Nine DERs were selected for qRT-PCR, and the expression profile of each miRNA was consistent with the sRNA-seq data (Figure 4), verifying the reliability of the sRNA results.

### miRNA Targets Identified Through Degradome-seq

In total, 84,600,973 RRs were obtained from both GM and YWt degradomes, representing 9,473,983 and 31,301,265 unique RRs, respectively (Supplementary Table 10). After removal of the 3′ adaptor and shorter cut label reads (<15 nt), 7,223,211 (76.24% of the unique mappable reads) and 5,795,795 (18.52%) unique reads were successfully mapped to 42,337 (75.82% of all 55,839 reference transcriptomes) and 41,195 (73.77%) single genes, respectively (Supplementary Table 10).

A total of 8346 target sites were identified for 571 miRNAs (Supplementary Figure 7); these included 3910 targets of 375 known miRNAs and 4436 targets of 196 novel miRNAs. In GM, 2389 (28.62%) targets belonged to category 0, 1, or 2, and 3644 targets were classified into category 3 or 4, while in YWt, 2019 (24.19%), the targets belonged to category 0, 1, or 2, and 3068 targets were classified into the other two categories (Supplementary Table 11). It is worth noting that a certain miRNA was involved in regulating multiple targets. For example, gma-miR396h, gma-miR6300, novel116_star, novel132_mature, novel20_star, novel59_mature, novel72_star, and osa-miR414 had more than 80 targets. In contrast, single genes are usually regulated by multiple miRNAs. For example, *TRINITY_DN8796_c0_g1_i1_2* may be regulated by aly-miR166b-5p, aly-miR396b-5p, gma-miR396h, and hbr-miR396a. In addition, some targets were annotated as TFs in *C. fortunei*, such as *APETALA2* (*AP2*) and *v-myb avian myeloblastosis viral oncogene homolog* (*MYB*), indicating that *C. fortunei* miRNAs may have the ability to target TF gene families.

### Enrichment Analyses of miRNA Targets

**FIGURE 5:**
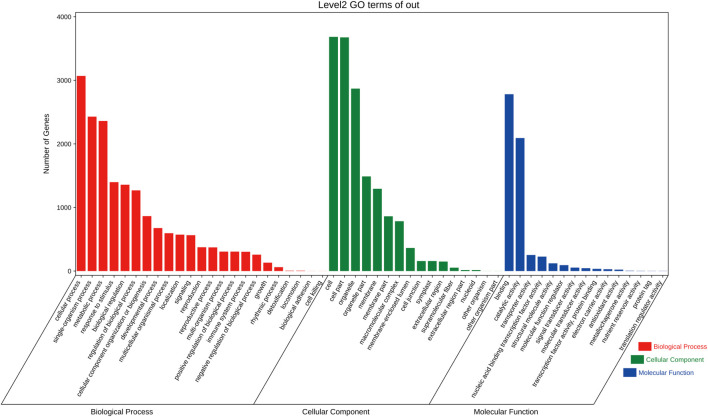
Gene Ontology classification of targets in *C. fortunei*. The *x*-axis is the entry name, and the *y*-axis represents the number of genes in the corresponding entry.

The functions of all targets were divided into three categories that included 54 GO terms by GO analysis. In BP, “cellular process,” “single-organism process,” and “metabolic process” had the highest number of target genes; in CC, the targets were mainly involved in “cell” and “cell part,” and in MF, the targets were mostly divided into “binding” and “catalytic activity” components (Figure 5).

Based on the KEGG analysis, these targets were matched to five categories: “genetic information processing,” “metabolism,” “organismal systems,” “cellular processes,” and “environmental information processing”; these categories represented 19 metabolic pathways. Among these pathways, “global and overview maps” (676), “carbohydrate metabolism” (254), and “translation” (239) were the most abundant (Supplementary Figure 8A). In addition, 72 and 48 targets were annotated to “metabolism of terpenoids and polyketides (TP)” and “biosynthesis of other specialized metabolites,” respectively (Supplementary Figure 8A). Notably, metabolism was the category with the highest number of annotated pathways. In metabolism, “genetic information processing and organismal systems,” “sesquiterpenoid and triterpenoid biosynthesis” (ko00909, 55.6%), “proteasome” (ko03050, 43.1%), and “circadian rhythm-plant” (ko04712, 48.9%) had the highest proportions of targets in each pathway (Supplementary Figure 8B).

### Comprehensive Analysis of TP

The metabolome, transcriptome, and miRNA target analyses described above showed that terpenoid and polyketide biosynthesis was significantly enriched. Therefore, we next focused on analyzing these metabolic pathways.

**FIGURE 6:**
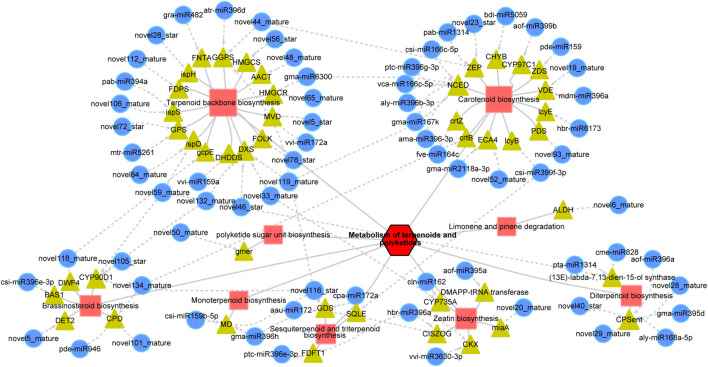
MicroRNA regulatory network involved in the metabolism of terpenoids and polyketides. The red squares indicate metabolic pathways, the yellow triangles indicate genes, and the blue circles indicate miRNAs. The dotted lines with arrows indicate how the miRNAs regulate specific genes. The abbreviation of each gene is as follows: *AACT*, acetyl-CoA C-acetyltransferase; *ALDH*, aldehyde dehydrogenase; *BAS1*, PHYB activation tagged suppressor 1; *CHYB*, beta-ring hydroxylase; *CISZOG*, *cis*-zeatin *O*-glucosyltransferase; *CKX*, cytokinin dehydrogenase; *CPD*, cytochrome P450 family 90 subfamily A polypeptide 1; *CPSent*, ent-copalyl diphosphate synthase; *crtB*, 15-*cis*-phytoene synthase; *crtZ*, beta-carotene 3-hydroxylase; *CYP735A*, cytokinin *trans*-hydroxylase; *CYP90D1*, 3-epi-6-deoxocathasterone 23-monooxygenase; *CYP97C1*, carotene epsilon-monooxygenase; *DET2*, steroid 5-alpha-reductase; *DHDDS*, ditrans, polycis-polyprenyl diphosphate synthase; *DMAPP-tRNA transferase*, tRNA dimethylallyltransferase; *DWF4*, steroid 22-alpha-hydroxylase; *DXS*, 1-deoxy-D-xylulose-5-phosphate synthase; *ECA4*, Ca^2+^-transporting ATPase; *FDFT1*, farnesyl-diphosphate farnesyltransferase; *FDPS*, farnesyl diphosphate synthase; *FNTA*, protein farnesyltransferase/geranylgeranyltransferase type-1 subunit alpha; *FOLK*, farnesol kinase; *GDS*, (–)-germacrene D synthase; *gcpE*, (E)-4-hydroxy-3-methylbut-2-enyl-diphosphate synthase; *gmer*, GDP-4-keto-6-deoxy-D-mannose-3,5-epimerase-4-reductase; *GGPS*, geranyl diphosphate synthase; *HMGCR*, hydroxymethylglutaryl-CoA reductase; *HMGCS*, hydroxymethylglutaryl-CoA synthase; *ispD*, 2-C-methyl-D-erythritol 4-phosphate cytidylyltransferase; *ispH*, 4-hydroxy-3-methylbut-2-en-1-yl diphosphate reductase; *ispS*, isoprene synthase; *lcyB*, lycopene beta-cyclase; *lcyE*, lycopene epsilon-cyclase; *MD*, (+)-neomenthol dehydrogenase; *miaA*, tRNA dimethylallyltransferase; *MVD*, diphosphomevalonate decarboxylase; *NCED*, 9-*cis*-epoxycarotenoid dioxygenase; *PDS*, 15-*cis*-phytoene desaturase; *GPS*, geranyl-diphosphate specific/all-*trans*-non-aprenyl-diphosphate synthase; *SQLE*, squalene monooxygenase; *VDE*, violaxanthin de-epoxidase; *ZDS*, zeta-carotene desaturase; *ZEP*, zeaxanthin epoxidase.

The miRNA targets related to the metabolism of TP mainly included nine main pathways, among which “terpenoid backbone biosynthesis,” “carotenoid biosynthesis,” and “diterpenoid biosynthesis” (Table 1) were more enriched; targets involved in “terpenoid backbone biosynthesis” (23, 31.94%), “carotenoid biosynthesis” (16, 22.22%), and “diterpenoid biosynthesis” (8, 11.11%) accounted for more than 65% of the total targets (Figure 6 and Table 1). We measured the levels of metabolites related to TP and found that except for the metabolites related to carotenoid biosynthesis, which were reduced in GM, most of the metabolites were significantly enhanced in GM (Table 2). In particular, in the diterpenoid biosynthesis pathway, the levels of gibberellin A14 and gibberellin A53 aldehydes exceeded the sum of the levels of the other 20 metabolites, and the levels of these metabolites in GM were 14.14 and 8.46 times those in YWt, respectively (Table 2).

**TABLE 1:** MicroRNAs target functions involved in the metabolism of terpenoids and polyketides.

Pathway	Gene number	Unigene	Gene
Terpenoid backbone biosynthesis	23	TRINITY_DN25002_c0_g2_i1_3	*atoB*
		TRINITY_DN26290_c0_g2_i2_2	
		TRINITY_DN22892_c0_g1_i8_1	*DHDDS*
		TRINITY_DN14808_c0_g1_i1_3	*DXS*
		TRINITY_DN24104_c0_g1_i15_1	
		TRINITY_DN26364_c0_g1_i1_3	
		TRINITY_DN11334_c0_g1_i2_3	*FDPS*
		TRINITY_DN23287_c0_g1_i4_1	*FNTA*
		TRINITY_DN26775_c0_g1_i15_2	
		TRINITY_DN22784_c0_g2_i1_3	*FOLK*
		TRINITY_DN28038_c0_g1_i4_2	*gcpE*
		TRINITY_DN14344_c0_g1_i1_3	*GGPS*
		TRINITY_DN17191_c0_g1_i1_1	
		TRINITY_DN25852_c0_g1_i1_2	
		TRINITY_DN14418_c0_g1_i1_3	*GPS*
		TRINITY_DN27103_c0_g1_i13_1	*HMGCR*
		TRINITY_DN27185_c0_g2_i2_3	*HMGCS*
		TRINITY_DN27344_c0_g2_i7_1	
		TRINITY_DN6885_c0_g1_i1_1	*ispD*
		TRINITY_DN26502_c0_g1_i2_1	*ispH*
		TRINITY_DN28637_c0_g1_i1_3	
		TRINITY_DN6836_c0_g1_i1_1	*ispS*
		TRINITY_DN9031_c0_g1_i1_3	*MVD*
Carotenoid biosynthesis	16	TRINITY_DN24797_c0_g1_i6_2	*CHYB*
		TRINITY_DN20337_c0_g1_i5_3	*crtB*
		TRINITY_DN23115_c0_g1_i2_1	*crtZ*
		TRINITY_DN18752_c0_g1_i2_3	*CYP97C1*
		TRINITY_DN20920_c0_g1_i1_1	*ECA4*
		TRINITY_DN21486_c0_g1_i1_1	*lcyB*
		TRINITY_DN27821_c0_g1_i8_2	*lcyE*
		TRINITY_DN28603_c0_g2_i1_2	*NCED*
		TRINITY_DN25721_c0_g1_i1_3	
		TRINITY_DN15115_c0_g1_i1_3	
		TRINITY_DN21999_c0_g1_i2_3	*PDS*
		TRINITY_DN7562_c0_g1_i1_3	*VDE*
		TRINITY_DN19004_c0_g1_i2_3	
		TRINITY_DN19208_c0_g1_i6_3	*ZDS*
		TRINITY_DN24472_c0_g1_i7_1	
		TRINITY_DN21855_c0_g1_i2_2	*ZEP*
Diterpenoid biosynthesis	8	TRINITY_DN25477_c0_g1_i1_3	*(13E)-labda-7,13-dien-17-ol synthase*
		TRINITY_DN26490_c0_g1_i9_3	
		TRINITY_DN27239_c0_g1_i2_2	
		TRINITY_DN27939_c0_g1_i4_3	
		TRINITY_DN29593_c0_g1_i2_2	*CPSent*
		TRINITY_DN29820_c0_g1_i21_2	
		TRINITY_DN30241_c0_g1_i2_2	
		TRINITY_DN30372_c1_g1_i15_2	
Brassinosteroid biosynthesis	8	TRINITY_DN29137_c0_g1_i5_2	*BAS1*
		TRINITY_DN21161_c0_g1_i2_3	*CPD*
		TRINITY_DN19646_c0_g1_i3_2	*CYP90D1*
		TRINITY_DN22770_c0_g1_i6_3	
		TRINITY_DN23701_c0_g1_i8_1	
		TRINITY_DN16116_c0_g1_i4_2	
		TRINITY_DN19591_c0_g1_i1_2	*DET2*
		TRINITY_DN31028_c0_g1_i1_2	*DWF4*
Zeatin biosynthesis	7	TRINITY_DN11266_c0_g1_i3_1	*CYP735A*
		TRINITY_DN14604_c0_g1_i2_3	*miaA*
		TRINITY_DN15706_c0_g1_i1_3	*CKX*
		TRINITY_DN18651_c0_g1_i4_3	
		TRINITY_DN26430_c1_g1_i4_3	*CISZOG*
		TRINITY_DN26700_c0_g1_i7_1	
		TRINITY_DN28118_c0_g3_i1_3	
Sesquiterpenoid and triterpenoid biosynthesis	5	TRINITY_DN23474_c0_g1_i1_1	*GDS*
		TRINITY_DN28996_c0_g1_i3_2	
		TRINITY_DN26528_c0_g1_i6_1	*FDFT1*
		TRINITY_DN30068_c0_g2_i4_2	
		TRINITY_DN25360_c0_g1_i1_3	*SQLE*
Monoterpenoid biosynthesis	3	TRINITY_DN26243_c0_g3_i8_1	*MD*
		TRINITY_DN30477_c0_g1_i4_2	
		TRINITY_DN30477_c0_g2_i2_2	
Polyketide sugar unit biosynthesis	1	TRINITY_DN26730_c0_g1_i3_2	*gmer*
Limonene and pinene degradation	1	TRINITY_DN17042_c0_g1_i2_3	*ALDH*

*atoB, acetyl-CoA C-acetyltransferase; *ALDH*, aldehyde dehydrogenase (NAD+); *BAS1*, PHYB activation tagged suppressor 1; *CHYB*, beta-ring hydroxylase; *CISZOG*, *cis*-zeatin *O*-glucosyltransferase; *CKX*, cytokinin dehydrogenase; *CPD*, cytochrome P450 family 90 subfamily A polypeptide 1; *CPSent*, ent-copalyl diphosphate synthase; *crtB*, 15-*cis*-phytoene synthase; *crtZ*, beta-carotene 3-hydroxylase; *CYP735A*, cytokinin *trans*-hydroxylase; *CYP97C1*, carotene epsilon-monooxygenase; *CYP90D1*, 3-epi-6-deoxocathasterone 23-monooxygenase; *DET2*, steroid 5-alpha-reductase; *DHDDS*, ditrans, polycis-polyprenyl diphosphate synthase; *DWF4*, steroid 22-alpha-hydroxylase; *DXS*, 1-deoxy-D-xylulose-5-phosphate synthase; *ECA4*, Ca^2+^-transporting ATPase; *FDFT1*, farnesyl-diphosphate farnesyltransferase; *FDPS*, farnesyl diphosphate synthase; *FNTA*, protein farnesyltransferase/geranylgeranyltransferase type-1 subunit alpha; *FOLK*, farnesol kinase; *gcpE*, (E)-4-hydroxy-3-methylbut-2-enyl-diphosphate synthase; *GDS*, (−)-germacrene D synthase; *GGPS*, geranylgeranyl diphosphate synthase; *gmer*, 3,5-epimerase/4-reductase; *GPS*, all-*trans*-non-aprenyl-diphosphate synthase; *HMGCR*, hydroxymethylglutaryl-CoA reductase (NADPH); *HMGCS*, hydroxymethylglutaryl-CoA synthase; *ispD*, 2-C-methyl-D-erythritol 4-phosphate cytidylyltransferase; *ispH*, 4-hydroxy-3-methylbut-2-en-1-yl diphosphate reductase; *ispS*, isoprene synthase; *lcyB*, lycopene beta-cyclase; *lcyE*, lycopene epsilon-cyclase; *miaA*, tRNA dimethylallyltransferase; *MD*, (+)-neomenthol dehydrogenase; *MVD*, diphosphomevalonate decarboxylase; *NCED*, 9-*cis*-epoxycarotenoid dioxygenase; *PDS*, 15-*cis*-phytoene desaturase; *SQLE*, squalene monooxygenase; *VDE*, violaxanthin de-epoxidase; *ZDS*, zeta-carotene desaturase; *ZEP*, zeaxanthin epoxidase.*

**TABLE 2:** Metabolites in terpenoids and polyketides.

Metabolite	Annotation	GM	YWt
Teasterone	Brassinosteroid biosynthesis	17.34 ± 5.32	1.08 ± 1.24
8′-Hydroxyabscisate	Carotenoid biosynthesis	1.18 ± 0.55	76.79 ± 17.82
Abscisic acid glucose ester		1.37 ± 0.77	0
Strigolactone ABC-rings		0.57 ± 0.88	34.42 ± 4.38
Xanthoxin		0.74 ± 0.90	20.59 ± 5.29
(+)-Sandaracopimaradiene	Diterpenoid biosynthesis	13.58 ± 2.17	1.93 ± 1.23
10-Deacetyl-2-debenzoylbaccatin III		43.41 ± 2.49	0.22 ± 0.08
10-Deacetylbaccatin III		11.63 ± 1.94	0
Gibberellin A12		49.99 ± 3.73	3.77 ± 2.29
Gibberellin A14		1894.47 ± 173.60	134.02 ± 28.60
Gibberellin A3		393.68 ± 39.33	52.30 ± 11.36
Gibberellin A34		16.65 ± 1.87	139.91 ± 5.77
Gibberellin A53 aldehyde		1298.52 ± 35.72	153.51 ± 35.72
Gibberellin A8-catabolite		21.34 ± 1.59	3.81 ± 2.63
Isopimaric acid		0	82.97 ± 28.49
Nocardicin C	Monobactam biosynthesis	355.91 ± 28.94	51.27 ± 13.83
(5R)-albaflavenol	Sesquiterpenoid and triterpenoid biosynthesis	43.78 ± 7.28	3.76 ± 3.78
(5S)-albaflavenol		27.25 ± 3.23	5.70 ± 3.83
Gossypol		36.40 ± 3.49	5.63 ± 0.32
All-*trans*-heptaprenyl diphosphate	Terpenoid backbone biosynthesis	282.89 ± 29.65	0
Coenzyme Q9	Ubiquinone and other terpenoid-quinone biosynthesis	56.60 ± 10.56	2.87 ± 2.82
Deoxyshikonin		36.04 ± 3.40	0.32 ± 0.49
Dihydrozeatin-O-glucoside	Zeatin biosynthesis	155.28 ± 5.57	12.50 ± 2.14

*The relative quantitative results (no unit) were calculated based on the peak area, and the values are the average of six replicate (*n* = 6) values. GM, #X1, evergreen mutant; YWt, #3, yellowish-brown needles in winter.*

**FIGURE 7:**
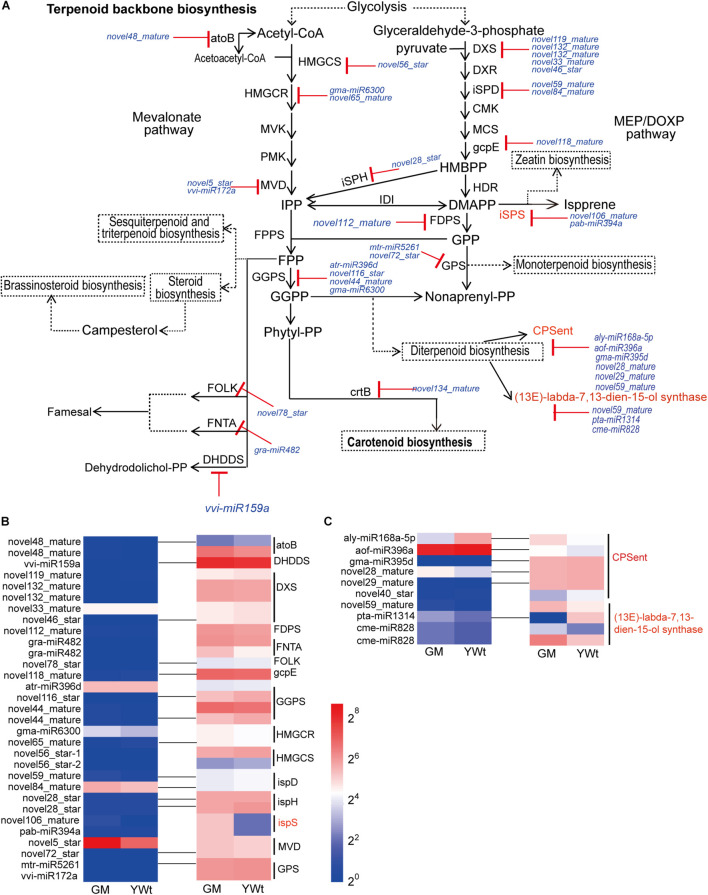
Analysis of miRNA targets involved in terpenoid and polyketide biosynthesis (TPB). **(A)** miRNA targets involved in TPB. miRNA cleavage is indicated by solid lines with red “T” arrows. miRNA targets involved in TPB **(B)** and diterpenoid biosynthesis **(C)**. The pseudo-color bar at the bottom of the middle represents the fragments per kilobase of exon per million (FPKM) value, GM, evergreen mutant; YWt, yellowish-brown needles in winter. The abbreviation of each gene/metabolite is as follows: *atoB*, acetyl-CoA acetyltransferase; *CMK*, 4-(cytidine 5-diphospho)-2-C-methyl-D-erythritol kinase; *CPSent*, ent-copalyl diphosphate synthase; *crtB*, 15-*cis*-phytoene synthase; *DHDDS*, ditrans, polycis-polyprenyl diphosphate synthase; *DMAPP*, dimethylallyl diphosphate; *DXR*, 1-deoxy-D-xylulose 5-phosphate reductoisomerase; *DXS*, 1-deoxy-D-xylulose-5-phosphate synthase; *FDPS*, farnesyl diphosphate synthase; *FNTA*, protein farnesyltransferase/geranylgeranyltransferase type-1 subunit alpha; *FOLK*, farnesol kinase; *FPP*, farnesyl diphosphate; *FPPS*, farnesyl pyrophosphate synthase; *gcpE*, (E)-4-hydroxy-3-methylbut-2-enyl-diphosphate synthase; *GGPP*, geranylgeranyl diphosphate; *GGPS*, geranylgeranyl diphosphate synthase; *GPP*, geranyl pyrophosphate; *HDR*, 4-hydroxy-3-methylbut-2-enyl diphosphate reductase; *HMBPP*, (E)-4-hydroxy-3-methyl-but-2-enyl pyrophosphate; *HMGCR*, hydroxymethylglutaryl-CoA reductase; *HMGCS*, hydroxymethylglutaryl-CoA synthase; *IDI*, isopentenyl diphosphate isomerase; *IPP*, isopentenyl diphosphate; *iSPD*, 2-C-methyl-D-erythritol 4-phosphate cytidylyltransferase; *iSPH*, 4-hydroxy-3-methylbut-2-en-1-yl diphosphate reductase; *iSPS*, isoprene synthase; *MCS*, 2-C-methyl-D-erythritol 2,4-cyclodiphosphate synthase; MEP/DOXP, 2-C-methyl-D-erythritol 4-phosphate/1-deoxy-D-xylulose 5-phosphate; *MVD*, diphosphomevalonate decarboxylase; *MVK*, mevalonate kinase; Non-aprenyl-PP, non-aprenyl pyrophosphate; Phytyl-PP, phytyl pyrophosphate; *PMK*, phosphomevalonate kinase; *GPS*, geranyl-diphosphate specific/all-*trans*-non-aprenyl-diphosphate synthase.

Based on these findings, we inferred that diterpenoid biosynthesis (including biosynthesis of the terpenoid backbone) is important in the formation of evergreen mutations in GM. Forty-one miRNA targets were related to terpenoid backbone and diterpenoid biosynthesis (Figure 7A). Thirty-one target pairs, including eight known miRNA targets [atr-miR396d-geranylgeranyl diphosphate synthase (*GGPS*), gma-miR6300-hydroxymethylglutaryl-CoA reductase (*HMGCR*), 2 gra-miR482-farnesyltransferase/geranylgeranyltransferase type-1 subunit alpha (*FNTA*), mtr-miR5261-geranyl-diphosphate specific/all-*trans*-non-aprenyl-diphosphate synthase (*GPS*), pab-miR394a-isoprene synthase (*ispS*), vvi-miR159a-ditrans, polycis-polyprenyl diphosphate synthase (*DHDDS*), and vvi-miR172a-diphosphomevalonate decarboxylase (*MVD*)], were related to terpenoid backbone biosynthesis. Among them, the DEG *ispS* (*TRINITY_DN6836_c0_g1_i1_1*) cleaved by novel106_mature and pab-miR394a was identified as participating in “isoprene synthesis” (Figure 7B). Twenty-three novel miRNA targets were related to terpenoid backbone biosynthesis, which included only contains three DERs (novel59_mature, novel106_mature, and novel5_star), and they were all highly expressed in GM; however, their targets were not significantly expressed (Figure 7B). Ten target pairs were identified as being involved in diterpenoid biosynthesis, namely, 6 *ent-copalyl diphosphate synthase* (*ent*) target genes cut by aly-miR168a-5p, aof-miR396a, gma-miR395d, novel28_mature, novel29_mature, and novel40_star and 4 *(13E)-labda-7,13-dien-15-ol synthase* target genes cut by novel59_mature, pta-miR1314, and cme-miR828 (Figure 7C). Among them, aly-miR168a-5p, aof-miR396a, and novel40_star were significantly upregulated (by 361.635, 29.540, and 20.516%, respectively) in YWt compared with GM, while others (gma-miR395d, novel28_mature, novel29_mature, novel59_mature, pta-miR1314, and cme-miR828) were significantly upregulated in GM (28.144–52.122%). It is worth noting that the targets of aly-miR168a-5p, aof-miR396a, novel59_mature, and cme-miR828 were significantly upregulated (by 40.144–163.608%) in GM compared with YWt.

To date, mainly six TF families [*AP2/ethylene responsive factor* (*ERF*), *basic helix-loop-helix* (*bHLH*), *MYB*, *NAM, ATAF, and CUC* (*NAC*), *WRKY* and *basic leucine zipper* (*bZIP*)] have been reported in plants in relation to the metabolism of terpenoids (Xu et al., 2019). In the study, only three TF families (12 *AP2*-like factors, 61 *MYBs*, and 2 *WRKYs*) were identified (Supplementary Table 12); among them, only five miRNAs (aly-miR172e-3p, cme-miR319c, novel137_mature, osa-miR159d, and zma-miR159e-3p) were differentially expressed. These findings suggest that these miRNA targets may play a vital role in differential diterpenoid biosynthesis and biological adaptation.

## Discussion

*Cryptomeria fortunei*, a very important industrial essential oil tree species in China, contains an especially large amount of natural terpenes (Yao et al., 2008; Xie et al., 2011). miRNAs play an important role in the life activities of plants by regulating gene expression (Bulgakov and Avramenko, 2015). In addition, a role for miRNAs in regulating plant specialized metabolites has been reported in an increasing number of plant species (Xu et al., 2013; Vashisht et al., 2015; Gupta et al., 2017; Liu et al., 2017). However, the possible regulatory role of *C. fortunei* miRNA in terpenoid biosynthesis is still unclear. Therefore, we used metabonome, transcriptome, sRNA, and degradome-seq to explore the potential miRNA regulatory network of terpenoids in *C. fortunei* needles, with the goal of improving the level of terpenoid synthesis in winter.

### Terpenoid Metabolites Were Differentially Produced in the Two Studied Phenotypes of *C. fortunei*

In *C. fortunei* needles, a total of 173 terpenoid metabolites were identified; these were primarily sesquiterpenoids but also included a variety of di-, mono-, and triterpenoids (Supplementary Table 2). Previous phytochemical studies of the branches, needles, heartwood, cones, and bark of *C. fortunei* have also isolated a variety of terpenoids, including mono-, sesqui-, and diterpenoids (Yao et al., 2008; Wu and Zhao, 2010), supporting the idea that terpenoids are present in large amounts in *C. fortunei*. However, there are obvious differences in the types and levels of compounds reported to be present. For example, Xie et al. (2011) identified only 68 terpenoids from the essential oil of *C. fortunei* using GC–MS. These differences in terpenoid metabolites may be related to the plant genetic background, environment, tissues/organs, sampling time, and many other factors.

We identified 31 terpenoids that were differentially biosynthesized in the two *C. fortunei* phenotypes analyzed in this study (Supplementary Table 3). Both metabolome and transcriptome functional analyses showed that DEGs/DSMs were enriched for diterpenoid biosynthesis (ko00904) (Figures 2, 3D). Therefore, we speculated that each of these two phenotypes of *C. fortunei* may have its own unique diterpenoid metabolic mechanisms. However, their specific biological regulatory mechanisms need to be further analyzed.

### miRNA TF Targets May Regulate Terpenoid Synthesis

A number of studies have shown that the production of terpenoid substances is strictly regulated by microribonucleic acids through TFs. We identified three TF families (*AP2*, *MYB*, and *WRKY*), most of which were *MYBs* and 35 *MYBs* were cut by miR828/858 (Supplementary Table 12). Other studies have also found that most of the target genes of miR858/828 belong to *MYBs*, especially *R2R3-MYB* TFs. For example, up to 20 *R2R3-MYBs* are involved in cotton fiber development (Guan et al., 2014), and most of them are related to the metabolic pathways of phenylpropanes. However, few studies have examined the synthesis of terpenoids. In loblolly pine (*Pinus taeda*), *PtMYB14* positively regulates the production of multiple sesquiterpenes (Bedon et al., 2010); in *A. thaliana*, *AtMYB* TFs increase terpenoid volatiles, not only by activating the transcription of the *trehalose-6-phosphate synthase* (*TPS*) gene but also by enhancing the metabolic flux to the isoprenoid pathway, while *MsMYB* negatively regulates the accumulation of monoterpenes and interferes with the synthesis of sesquiterpenes and diterpene derivatives in spearmint (Reddy et al., 2017). Therefore, whether a specific *MYB* regulates terpenoid synthesis and how to regulate it (e.g., negative/positive regulation, which structural gene to regulate) remains to be further studied. In addition, in *Artemisia annua*, the *AP2* TFs *AaERF1/2* participate in actively regulating expression of the sesquiterpene synthase gene (Yu et al., 2012). In Asian cotton (*Gossypium arboreum*), *GaWRKY1* activates the transcription of the sesquiterpene synthase gene encoding cadinene synthase (*CAD1*), which leads to the biosynthesis of sesquiterpene gossypol (Xu et al., 2004), which is similar to our research results. Other TFs have also been found to be involved in the biosynthesis of terpenes. For example, in *Arabidopsis*, miR156 regulates the content of active ingredients such as sesquiterpenes by downregulating its target gene *SPL9* (Yu et al., 2015). Therefore, it is still necessary to enrich the sample tissues and the cedar leaves of different growth stages in follow-up studies to further explore the mechanism by which miRNA-TF regulates terpenoid biosynthesis.

### miRNA Target Genes Play an Important Role in Regulating Diterpenoid Synthesis

**FIGURE 8:**
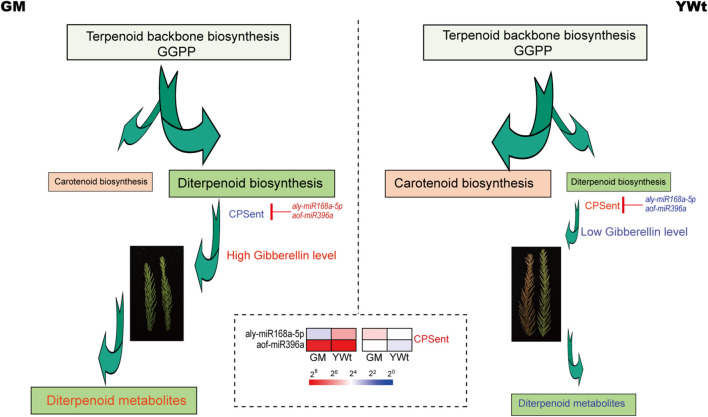
Simplified model of aly-miR168a-5p- and aof-miR396a-CPSent regulating diterpenoid biosynthesis. Red/blue text indicates up-/downregulated genes; the sizes of the arrows indicate the amount of metabolic flow; and pseudo-color bar at the bottom of the middle represents the fragments per kilobase of exon per million (FPKM) value. *CPSent*, ent-copalyl diphosphate synthase; *GGPP*, geranylgeranyl diphosphate; GM, evergreen mutant; YWt, yellowish-brown needles in winter.

MicroRNAs usually partially or completely match their target genes, leading to endonuclease digestion or translation inhibition and thereby regulate the expression of the target genes and play important roles. Therefore, searching for target genes is of great value for elucidating the biological functions of miRNAs. Degradome-seq, which offers high throughput and coverage, has been suggested to significantly increase the probability of capturing target miRNA fragments and to provide a statistically significant number of all target sites for each miRNA (Pantaleo et al., 2010). It has therefore been widely used in research on plant miRNA targets (Xu et al., 2013; Chen et al., 2019; Ye et al., 2020). miRNA targets were identified by degradome-seq; through GO and KEGG analysis, these miRNA targets were found to be involved in a variety of molecular biological regulatory pathways, including terpenoid and polyketide biosynthesis (Figure 5 and Supplementary Figure 8). Further miRNA target and metabolite analysis showed that terpenoid backbone biosynthesis, diterpenoid biosynthesis and carotenoid biosynthesis play a vital role in terpenoid biosynthesis in *C. fortunei* (Figure 6 and Tables 1, 2). In addition, most of the orange-color-related DSMs in carotenoid biosynthesis were significantly upregulated in YWt, and most of the remaining terpenoid and polyketide biosynthesis-related metabolites were upregulated in GM (Table 2). As a common precursor, geranylgeranyl diphosphate (GGPP) has three main routes of metabolism: (1) *via* a reaction catalyzed by *phytoene synthase* (*PSY*), GGPP is further condensed to phytoene (C40) and participates in carotenoid biosynthesis; (2) *via* a reaction catalyzed by *geranylgeranyl reductase* (*GGR*), GGPP eventually forms the phytol side chain of chlorophyll; and (3) *via* a reaction catalyzed by *diterpene synthase*, various diterpene compounds, including gibberellin A and some phytoalexins, are formed. Therefore, we inferred that in *C. fortunei* terpenoid backbone biosynthesis, the precursor GGPP is allocated to different metabolic branches, such as carotenoid and diterpenoid biosynthesis; that is, YWt contains more carotenoids than GM, and GM contains more diterpenoids than YWt, resulting in differences in needle color between GM and YWt due to differential synthesis of diterpenoids (Figure 8).

One miRNA can regulate hundreds of targets, and one target can also be regulated by multiple miRNAs (Licursi et al., 2019), thus constructing a complex interlocking miRNA-target regulatory network (Figure 6). Among them, we identified 41 miRNA targets synthesized *via C. fortunei* terpenoid backbone biosynthesis and diterpenoids. For many modules, the expression pattern of miRNA was opposite to that of its target genes, indicating that these miRNAs could negatively regulate their targets (Figures 7B,C). In terpenoid backbone biosynthesis, we identified multiple known miRNAs (atr-miR396d, gma-miR6300, gra-miR482, mtr-miR5261, pab-miR394a, vvi-miR159a, and miR172a) that regulated structural gene (such as *GGPS*, *HMGCR*, and *FNTA*) (Figure 7A). In Chinese toon (*Toona sinensis*) sprouts, tsi-miR408-3p, tsi-miR171a, tsi-miR390b-5p, and tsi-miR-1260 target these unigenes, such as *HMGCR*, *MEP*, and *1-deoxy-D-xylulose-5-phosphate synthase* (*DXS*) (Zhao et al., 2021); only three miRNA-enzyme gene pairs related to terpenoid biosynthesis have been identified in tea trees (*Camellia sinensis*) (Zhao et al., 2018). There are some differences in miRNA targets, which may be related to species specificity, but these also confirm that miRNAs are indeed involved in the biosynthesis of terpenoids. We also identified multiple novel miRNA targets in this pathway (Figure 7A), which is similar to the phenomenon in *T. sinensis* sprouts (Zhao et al., 2021). Novel miRNAs are a type of speculative miRNA, which indicates that these genes may be the basis of specific biological functions in *C. fortunei*, and further experiments are needed to determine the functions of these novel miRNAs. Interestingly, the differentially expressed target gene *ispS* (*TRINITY_DN6836_c0_g1_i1_1*) cut by novel106_mature and pab-miR394a was identified as participating in “isoprene synthesis” (Figure 7). The interpretation of miRNA regulatory functions is inseparable from its target genes. In land plants, the target gene *ispS* is located in the chloroplast (Sasaki et al., 2005), where it catalyzes the conversion of the intermediate substrate DMAPP to isoprene (Silver and Fall, 1991). In our previous research, we found that the chloroplasts in GM were basically intact, whereas the chloroplast structure in YWt was destroyed in winter. Therefore, it was also confirmed that the synthesis of *ispS* was indeed inhibited in YWt. Previous studies have shown that isoprene, a volatile C5 terpenoid compound, is synthesized by plants and that the biological release of isoprene by plants has a certain protective effect (Kksal et al., 2010; Oku et al., 2015). Therefore, we inferred that GM could produce more isoprene to enhance its adaptability to low temperatures.

In the diterpenoid biosynthesis modules, that is, in aly-miR168a-5p- and aof-miR396a-*CPSent* and in novel59_mature- and cme-miR828-*(13E)-labda-7,13-dien-15-ol synthase* (*CPSKSL1*), the target genes were upregulated in GM (Figure 7C). *CPSent* is a key enzyme in the gibberellin biosynthesis pathway in plants (Otomo et al., 2004) and in the phytoalexin synthesis pathway. It catalyzes the cyclization of GGDP to ent-copalyl diphosphate (CDP) (Xu et al., 2010). Gibberellin is a very important plant hormone in higher plants. It regulates stem elongation, leaf expansion, flower differentiation and seed and fruit development, breaks dormancy, promotes germination, reduces organ shedding, and participates in many other physiological processes (Lang, 1957). Phytoalexins are small-molecular-weight compounds with antimicrobial activity that are synthesized and rapidly accumulate in the body when plants are attacked by biological or non-biological threats. The phytoalexins that have been isolated and identified to date can be roughly divided into terpenes, flavonoids, flavanones, and alkaloids. The phytoalexin components of these diterpenoids enhance the disease resistance of plants and their resistance to related exogenous damage to some extent. Therefore, we inferred that compared with YWt, the low expression of aly-miR168a-5p and aof-miR396a in GM promoted high expression of its target *CPSent* genes (*TRINITY_DN30372_c1_g1_i15_2* and *TRINITY_DN29820_c0_g1_i21_2*), which in turn promoted the accumulation of gibberellins such as gibberellin A12, A14, and A3, gibberellin A53 aldehyde and gibberellin A8 catabolite (Figure 8). Therefore, GM exhibited reduced dormancy and remained green, a condition that ensured that it could produce more diterpenoids (Figure 8). The bifunctional *CPSKSL1* gene product catalyzes the formation of the inner double bond isomer of cobalt cyclopentadiphosphate (CPP) and subsequently replaces diphosphate with a hydroxyl group to form labda-7,13E-dien-15-ol (Mafu et al., 2011). Although the latter compound is a known plant metabolite, its biosynthesis has not yet been studied, and further experimental verification is needed.

### miRNA Targets Play a Role in Regulating Flavonoid Biosynthesis to Some Extent

Differentially synthesized metabolites, DEGs, and miRNA targets were all significantly enriched in the flavonoid metabolism pathway (Figures 2, 3D and Supplementary Figure 9). (+)-Catechin, epicatechin, luteoforol, and pentahydroxyflavanone were significantly upregulated in YWt; in that phenotype, the level of epicatechin was 11.81-fold higher than that in GM and much higher than the levels of other metabolites; afzelechin, homoeriodictyol, and pinocembrin were significantly upregulated in GM (Supplementary Table 13). These results showed that the two phenotypes of *C. fortunei* exhibited significant differences in flavonoid metabolism.

A total of 22 miRNA targets were related to flavonoids (Supplementary Figure 9). We further selected target pairs with different target genes, that is, ata-miR395b-3p-*flavonol synthase* (*FLS*, *TRINITY_DN24453_c0_g1_i3_1*, and *TRINITY_DN29464_c0_g1_i4_2*), novel116_star-*chalcone synthase* (*CHS*, *TRINITY_DN26891_c0_g2_i2_3*), novel20_star-*flavanone 3-hydroxylase gene* (*F3H*, *TRINIT Y_DN19610_c1_g1_i2_1*), novel55_mature-*leucoanthocyanidin dioxygenase* (*LDOX*, *TRINITY_DN27808_c0_g1_i2_3*), and lja-miR398-3p-*leucoanthocyanidin reductase* (*LAR*, *TRINITY_DN23847_c0_g1_i4_1*), and we found that only *LAR* was upregulated in GM, whereas the remaining target genes were upregulated in YWt. Studies have shown that miR395 acts on target genes and negatively regulates the key flavonoid gene *FLS*, which ultimately affects the accumulation of flavonoids (Li et al., 2018). Therefore, we believe that *FLS* is highly expressed in YWt and that this expression promotes flavonoid accumulation. Novel116_star, novel20_star, and novel55_mature are novel predicted miRNAs, with 204, 126, and 16 predicted targets, respectively, most of which are unknown. These genes showed no homology with genes from other species, indicating that they may support specific functions in *C. fortunei*. In addition, few studies have examined the role of miR398 in regulating flavonoid biosynthesis, and further experiments are needed to determine the functions of these miRNAs.

## Conclusion

In summary, we analyzed the transcriptome, metabolome, sRNA, and degradome of two different overwintering *C. fortunei* phenotypes. The results of the metabolome and transcriptome analyses showed that DSMs and DEGs were enriched in the diterpenoid pathway. The miRNAs and their targets in *C. fortunei* needles were then identified, and the miRNA targets involved in terpenoid biosynthesis were analyzed. The module (aly-miR168a-5p- and aof-miR396a-*CPSent*) may be a potential key factor in the mechanism of differential diterpenoid synthesis in winter. In addition, we also identified three miRNA-TF families, that may be related to terpenoid biosynthesis, but further experimental verification is required. These findings provide reference information regarding diterpenoid regulatory mechanisms in *C. fortunei* and valuable resources for bioengineering research related to plant production of essential oils in winter.

## Data Availability Statement

The datasets presented in this study can be found in online repositories. The names of the repository/repositories and accession number(s) can be found below: https://www.ncbi.nlm.nih.gov/genbank/, PRJNA697258; https://www.ncbi.nlm.nih.gov/genbank/, PRJNA720228.

## Author Contributions

JX conceived and designed the experiments. JX and YZ performed the experiments and wrote the manuscript. JC, HH, JYX, and JY analyzed the data. All authors read and approved the final manuscript.

## Conflict of Interest

The authors declare that the research was conducted in the absence of any commercial or financial relationships that could be construed as a potential conflict of interest.

## Publisher’s Note

All claims expressed in this article are solely those of the authors and do not necessarily represent those of their affiliated organizations, or those of the publisher, the editors and the reviewers. Any product that may be evaluated in this article, or claim that may be made by its manufacturer, is not guaranteed or endorsed by the publisher.

## Footnotes

1
https://cloud.oebiotech.cn/task/

2
http://www.sanger.ac.uk/software/Rfam

3
http://www.ncbi.nlm.nih.gov/genbank/

4
http://www.mirbase.org/

5
https://www.omicshare.com/

6
https://cytoscape.org/

## Abbreviations

AP2: APETALA2
BP: biological process
CC: cellular component
*CPSent*: *ent-copalyl diphosphate synthase*
CRs: clean reads
CSRs: cleavage site reads
DEGs: differentially expressed unigenes
DSMs: differentially synthesized metabolites
DERs: differentially expressed miRNAs
GGPP: geranylgeranyl diphosphate
GO: Gene Ontology
*ispS*: *isoprene synthase*
KEGG: Kyoto Encyclopedia of Genes and Genomes
LC–MS: liquid chromatography-mass spectrometry
MF: molecular function
miRNAs: microRNAs
MYB: v-myb avian myeloblastosis viral oncogene homolog
(O)PLS-DA: (orthogonal) partial least squares-discriminant analysis
qRT-PCR: quantitative real-time polymerase chain reaction
RRs: raw reads
sRNA: small RNA
TFs: transcription factors
TP: terpenoids and polyketides
VIP: variable importance in the projection.
