# Supplementary material for: Integrated Four Comparative-Omics Reveals the Mechanism of the Terpenoid Biosynthesis in Two Different Overwintering Cryptomeria fortunei Phenotypes
Source: Front Plant Sci. 2021 Sep 29;12:740755. doi: 10.3389/fpls.2021.740755 (PMC8513690; doi:10.3389/fpls.2021.740755)
Supplement: Supplementary file 1 [file Data_Sheet_1.docx]

Supplementary Material

## Supplementary Figures

**
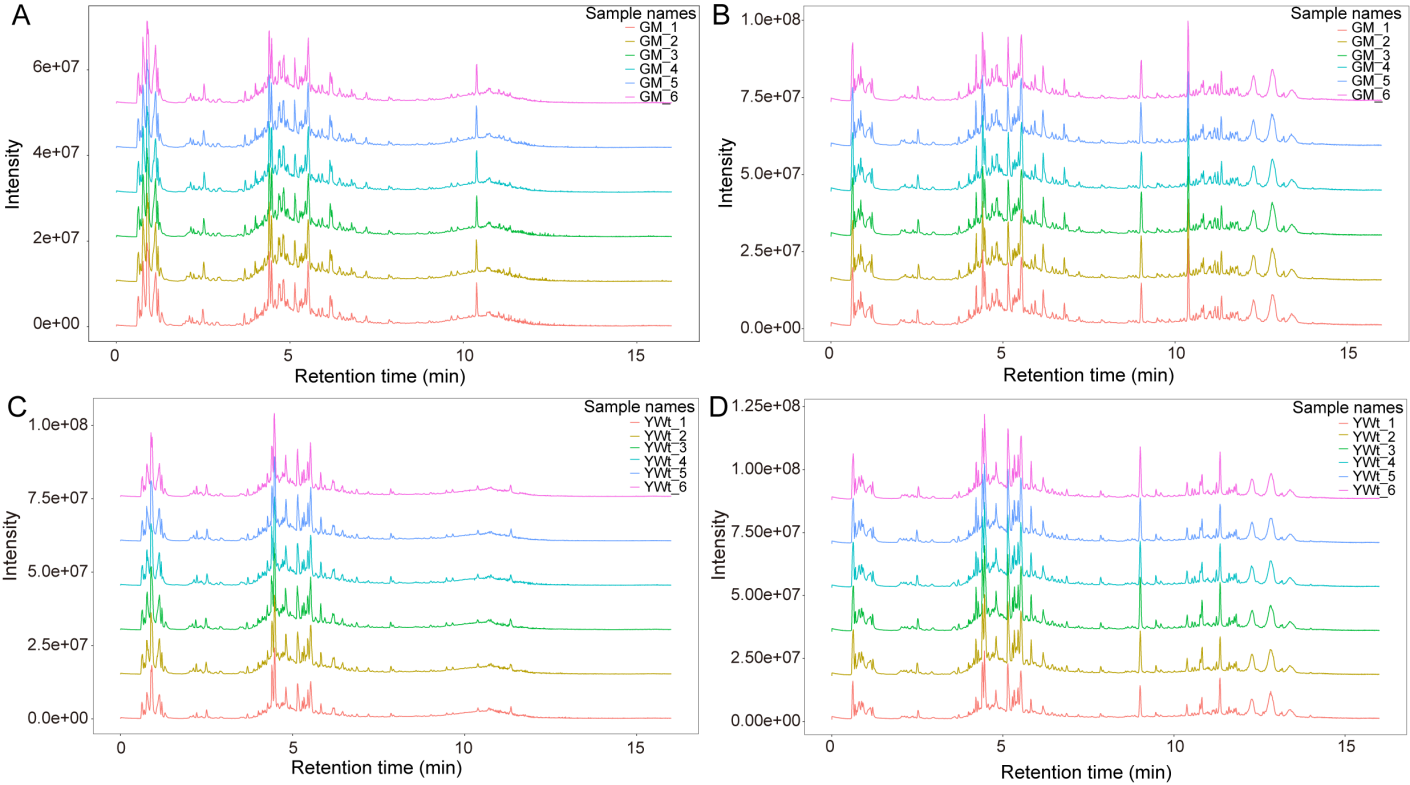
**

**Supplementary Figure 1.** Total ion current (TIC) plots for 12 samples. Analysis of GM under negative **(A)** and positive **(B)** ion modes. Analysis of YWt in negative **(C)** and positive **(D)** ion modes. The *x*- and *y*-axes represent retention time and intensity, respectively. GM, evergreen mutant; YWt, yellowish-brown needles in winter. In each plot, the sample names indicate the 6 biological repeats (n = 6, labelled GM_1, GM_2, GM_3, GM_4, GM_5 and GM_6) measured; each is displayed in its own color.

**
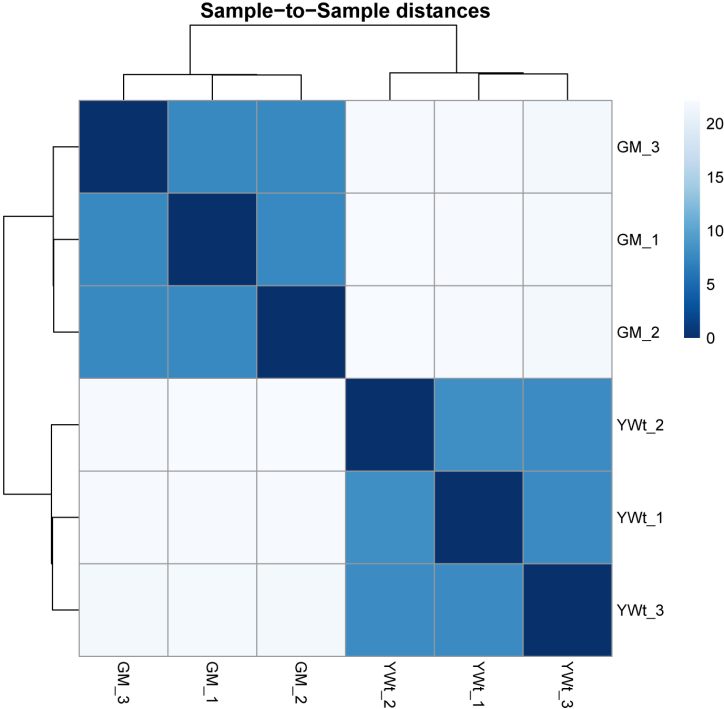
Supplementary Figure 2.** Cluster analysis of samples based on transcriptome data. The *x*- and *y*-axes both represent samples, and the pseudo-color bar on the top right represents the Euclidean distance between the samples. GM, evergreen mutant; YWt, yellowish-brown needles in winter. GM_1, GM_2 and GM_3 represent 3 repetitions.


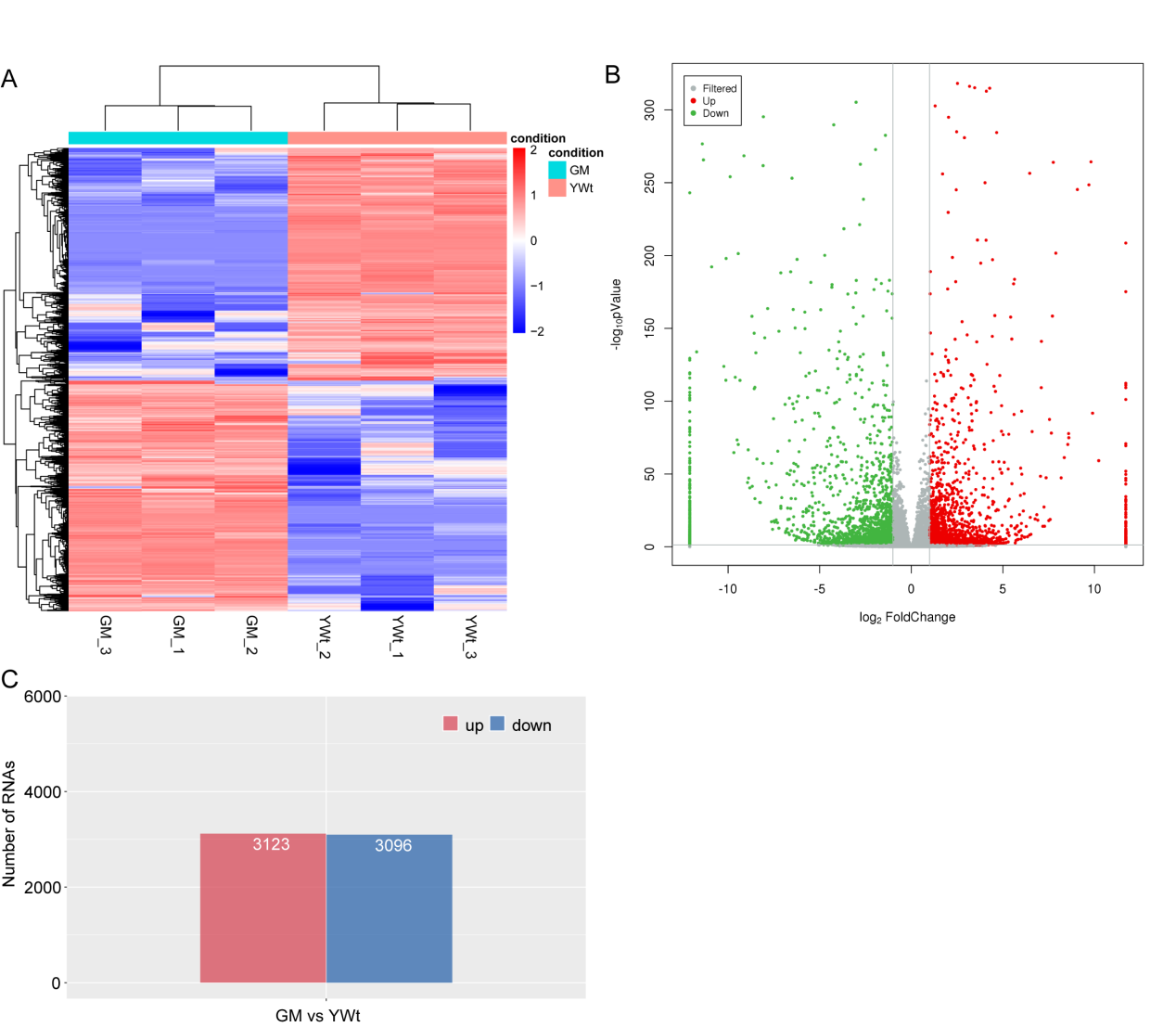
**Supplementary Figure 3.** Differentially expressed unigene (DEG) analysis of the transcriptome. **(A)** Hierarchical clustering heatmap of DEGs. Each column and row represent a sample and a DEG, respectively, and the colors indicate the expression levels of the genes; that is, the redder the color is, the higher is the expression level. **(B)** Volcano map of DEGs. The red and green dots represent significantly up- and downregulated DEGs, respectively, while the grey dots represent nonsignificantly changed DEGs. **(C)** Histogram of the number of DEGs. The *y*-axis represents the number of DEGs. GM, evergreen mutant; YWt, yellowish-brown needles in winter. GM_1, GM_2 and GM_3 represent 3 repetitions.

**
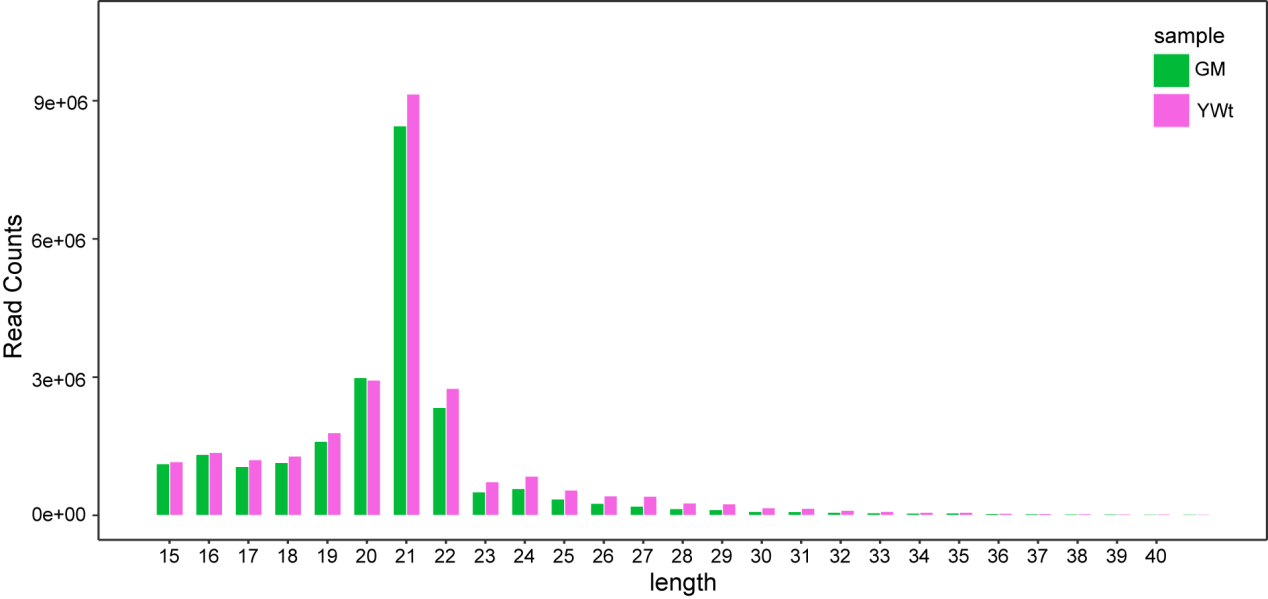
Supplementary Figure 4.** Length distribution of miRNAs. The *x*- and *y*-axes represent the length of miRNAs and the read counts of miRNAs, respectively. Different colors indicate different samples (GM, evergreen mutant; YWt, yellowish-brown needles in winter).


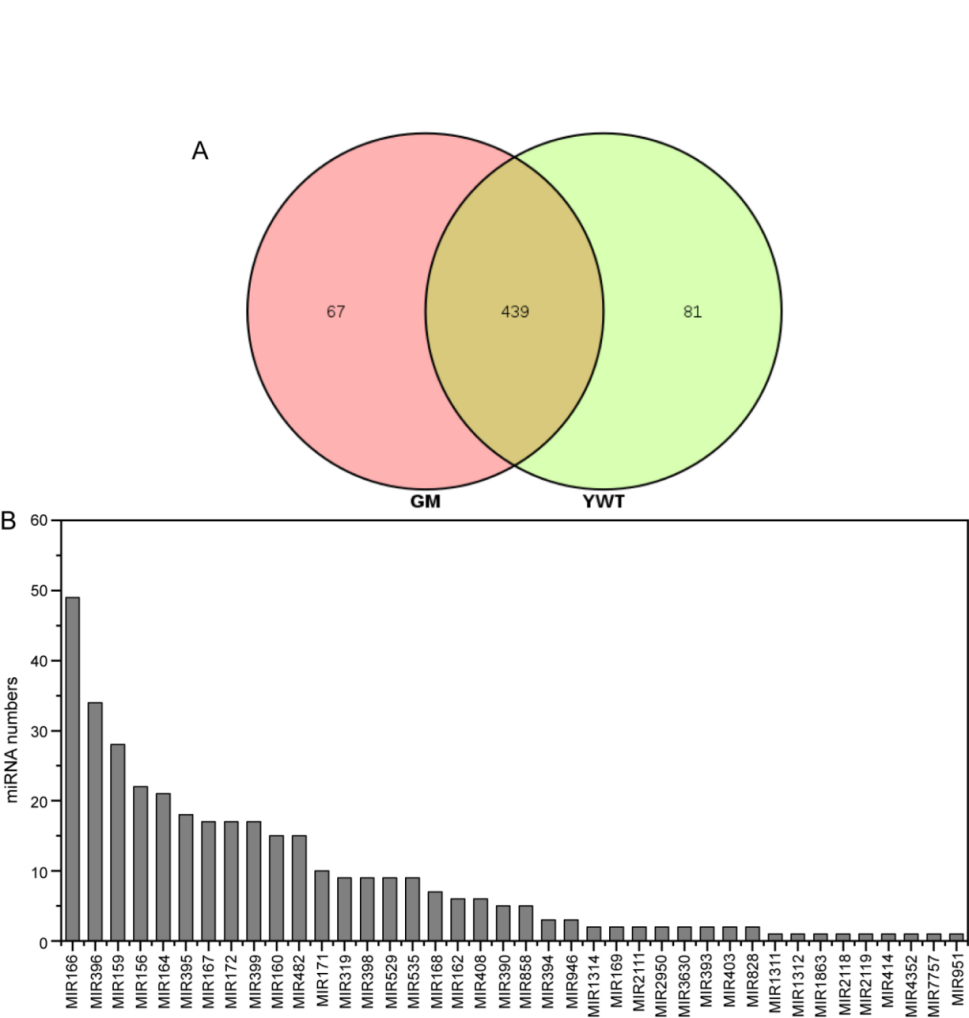
**Supplementary Figure 5.** Analysis of the number of miRNAs. **(A)** Number of miRNAs detected in the two samples. GM, evergreen mutant; YWt, yellowish-brown needles in winter. **(B)** MiRNA family analysis. The *x*- and *y*-axes represent miRNA families and miRNA numbers, respectively.


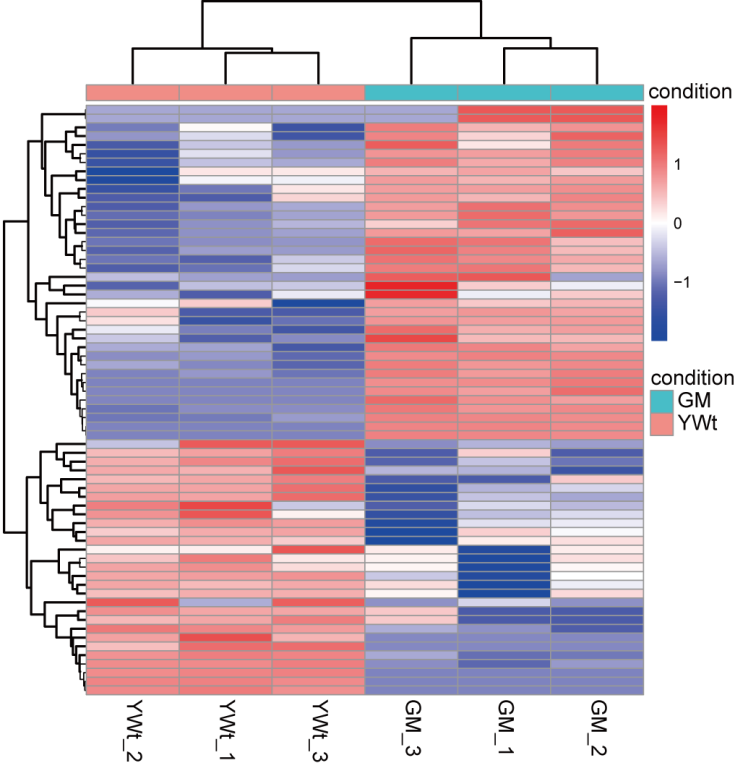
**Supplementary Figure 6.** Analysis of differentially expressed miRNAs (DERs). **(A)** Hierarchical clustering heatmap of DERs. Each column and each row represent a sample and a DER, respectively, and the colors indicate the expression levels of the DERs. GM, evergreen mutant; YWt, yellowish-brown needles in winter. GM_1, GM_2 and GM_3 represent 3 repetitions.


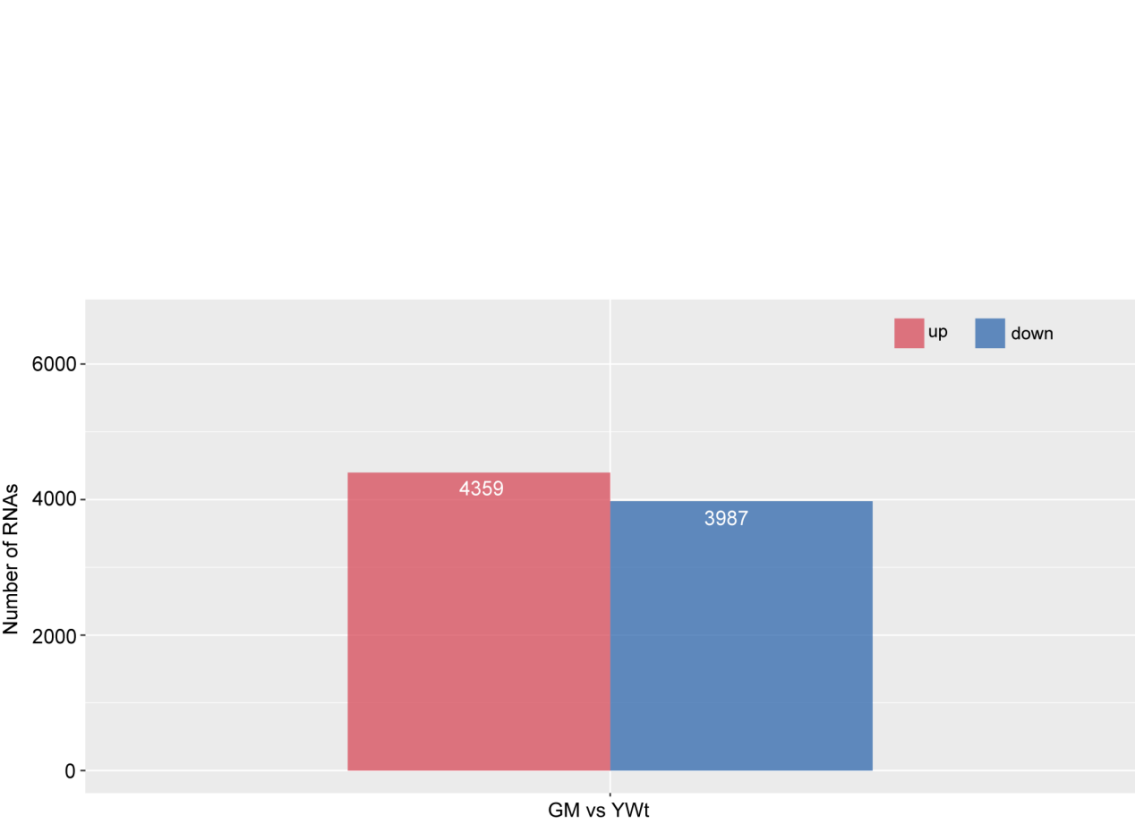
**Supplementary Figure 7.** Analysis of the number of miRNA targets. The *y*-axis represents the number of miRNA targets. GM, evergreen mutant; YWt, yellowish-brown needles in winter.


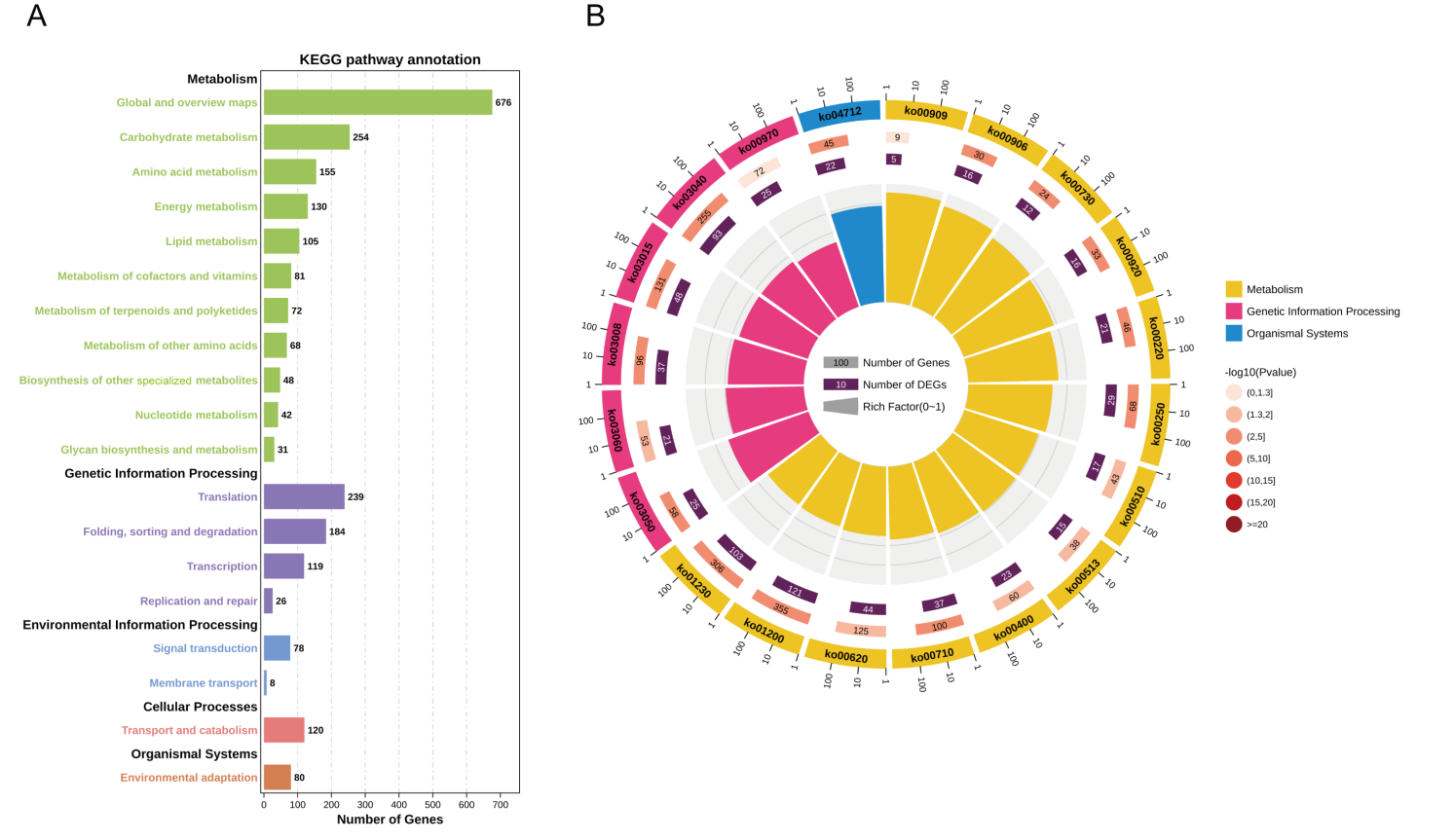


**Supplementary Figure 8.** KEGG annotation information for miRNA targets in *C. fortunei*. **(A)** KEGG annotation of miRNA targets. The *y*-axis represents the KEGG pathway, and the *x*-axis indicates the number of targets annotated to each pathway. **(B)** Circle plot of KEGG enrichment analysis of miRNA targets. There are four circles from the outside to the inside: the enriched category, the number and q-value of the category in the background genes, the ratio of differentially expressed genes and the rich factor value of each category.


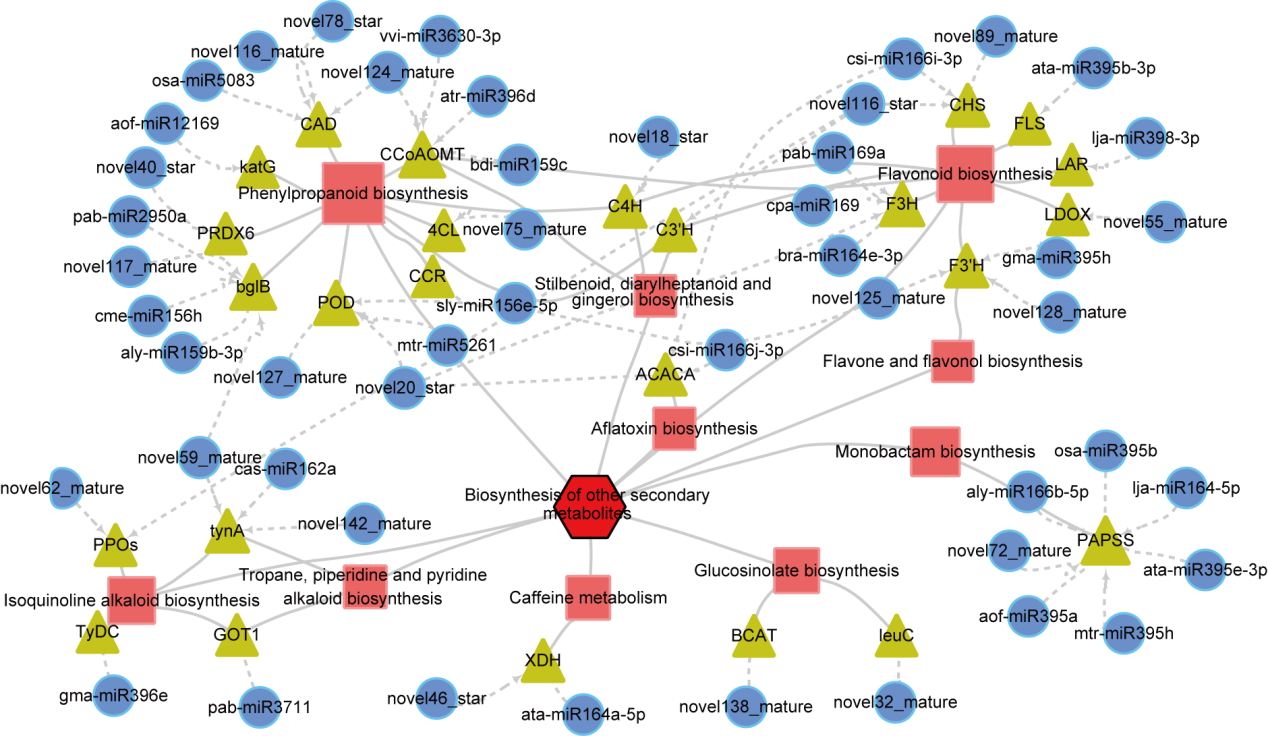
**Supplementary Figure 9.** MiRNA regulatory network involved in the biosynthesis of other specialized metabolites. The red squares indicate metabolic pathways, the yellow triangles indicate genes, and the blue circles indicate miRNAs. The dotted lines with arrows indicate how the miRNAs regulate specific genes. The abbreviation of each gene is as follows: *4CL*, 4-coumarate-CoA ligase; *ACACA*, acetyl-CoA carboxylase/biotin carboxylase 1; *BCAT*, branched-chain amino acid aminotransferase; *bglB*, beta-glucosidase; *C3'H*, coumaroylquinate(coumaroylshikimate) 3'-monooxygenase; *C4H*, trans-cinnamate 4-monooxygenase; *CAD*, cinnamyl-alcohol dehydrogenase; *CCoACMT*, caffeoyl CoA 3-O-methyltransferase; *CCR*, cinnamoyl-CoA reductase; *CHS*, chalcone synthase; *F3H*, naringenin 3-dioxygenase; *F3'H*, flavonoid 3'-monooxygenase; *FLS*, flavonol synthase; *GOT1*, glutamate oxaloacetate transaminase 1; *katG*, catalase-peroxidase; *LAR*, leucoanthocyanidin reductase; *LDOX*, leucoanthocyanidin dioxygenase; *leuC*, 3-isopropylmalate/(R)-2-methylmalate dehydratase large subunit; *PAPSS*, 3'-phosphoadenosine 5'-phosphosulfate synthase; *POD*, peroxidase; *PPOs*, polyphenol oxidase; *PRDX6*, peroxiredoxin 6, 1-Cys peroxiredoxin; *TyDC*, tyrosine decarboxylase; *tynA*, primary-amine oxidase; *XDH*, xanthine dehydrogenase/oxidase.

**Supplementary Tables**

**Supplementary Table 1.** Primer sequences.

| **miRNA** | **Primer sequence (5’ to 3’)** |
| --- | --- |
| aly-miR166a-3p | GGACCAGGCTTCATTCCCC |
| ata-miR166c-3p | CGGACCAGGCTTCATTCCTT |
| cca-miR396c | CGTTCAAGAAAGCTGTGGGAAAA |
| aly-miR399b-3p | GCCAAAGGAGAGTTGCCCTG |
| pab-miR11534 | CGTGAGATTGTTGGAGAGGTTCA |
| novel5_star | CGAATGGGCTGATTGACAAAACC |
| novel19_mature | GCTCCCAGGTTGAGAGGAATCT |
| novel38_mature | CGCTGACTGTTATCTGTGTGAGAG |
| novel45_mature | CGGTTGTAGCCTGGATAATCTCTTA |
| cln-miR6725 | TGGCATCTGTCGAGGTCATCTA |
| *U6* | Forward: ACAGAGAAGATTAGCATGGCC |
|  | Reverse: GACCAATTCTCGATTTGTGCG |

**Supplementary Table 2.** Terpenoid-related metabolites in *C. fortunei*.

| **Subclass** | **Metabolite** | **GM** | **YWt** |
| --- | --- | --- | --- |
| diterpenoids | ent-kaur-16-en-19-al | 263.016 | 163.216 |
|  | Carnosic acid | 3079.327 | 87.702 |
|  | Yucalexin P15 | 419.378 | 91.697 |
|  | Gibberellin A87 | 181.523 | 261.647 |
|  | Retapamulin | 184.245 | 40.993 |
|  | Erinacine C | 2802.747 | 3349.102 |
|  | Crocin 3 | 420.704 | 417.702 |
|  | 11-[(2R)-3-[2-amino-3-methyl-4-(2-methyl-1,3-thiazol-4-yl)but-3-en-1-yl]-2-methyloxiran-2-yl]-3-hydroxy-4,4,6,8-tetramethyl-5-oxo-7-(sulfooxy)undecanoic acid | 91.909 | 125.834 |
|  | Ecabet | 1822.539 | 1690.036 |
|  | Dicrocin | 843.254 | 697.047 |
|  | Cincassiol B | 5.958 | 6.108 |
|  | Gibberellin A124 | 208.380 | 360.005 |
|  | ent-16b,19-Kauranediol 19-acetate | 50.025 | 7.782 |
|  | Kaur-16-en-18-al | 151.574 | 88.060 |
|  | Tricrocin | 287.029 | 4.507 |
|  | 11beta-Hydroxygibberellin A7 | 22.269 | 2.124 |
|  | Gibberellin A38 | 45.816 | 34.149 |
|  | Crocin 4 | 21.606 | 87.115 |
|  | Abietinal | 77.259 | 60.951 |
|  | 11-[(2R)-3-[2-amino-3-methyl-4-(2-methyl-1,3-thiazol-4-yl)but-3-en-1-yl]-2-methyloxiran-2-yl]-7-hydroxy-4,4,6,8-tetramethyl-5-oxo-3-(sulfooxy)undecanoic acid | 147.211 | 108.325 |
|  | 12-Hydroxy-11-methoxy-8,11,13-abietatrien-20-oic acid | 191.190 | 10.442 |
|  | Siderol | 152.726 | 17.569 |
|  | (3b,4b,11b,14b)-11-Ethoxy-3,4-epoxy-14-hydroxy-12-cyathen-15-al 14-xyloside | 208.436 | 351.950 |
|  | Isopimaric acid | 0.000 | 82.973 |
|  | 11-[(2R,3S)-3-[2-amino-3-methyl-4-(2-methyl-1,3-thiazol-4-yl)but-3-en-1-yl]-2-methyloxiran-2-yl]-3,7-dihydroxy-4,4,6,8-tetramethyl-5-oxoundecanoic acid | 38.113 | 17.030 |
|  | Erinacine E | 30.418 | 18.207 |
|  | Gibberellin A110 | 35.382 | 17.031 |
|  | Geranylgeranylcysteine | 446.084 | 865.361 |
|  | Gibberellin A39 | 5.407 | 0 |
|  | Cavipetin A | 0 | 66.822 |
|  | Armillane | 72.535 | 82.638 |
|  | 3,3'-Bisanigorufone | 32.966 | 27.026 |
|  | (12S,15S)-15-O-Demethyl-10,29-dideoxy-11,12-dihydro-striatin C | 37.092 | 25.313 |
|  | Annoglabasin C | 6.220 | 18.308 |
|  | 3,3'-Bis(4''-hydroxyanigorufone) | 4.174 | 0 |
| monoterpenoids | Valtrate | 388.250 | 234.878 |
|  | 5-Isopropyl-2-(2-methylpropyl)-2-cyclohexen-1-one | 329.392 | 343.400 |
|  | (1beta,2beta,5beta)-p-Menth-3-ene-1,2,5-triol | 300.915 | 350.993 |
|  | Helinorbisabone | 18.890 | 7.725 |
|  | 10-Isopropyl-2,7-dimethyl-1-oxaspiro[4.5]deca-3,6-diene | 91.023 | 109.340 |
|  | (1R,2S,3S,4R)-p-Menthane-2,3-diol | 50.700 | 29.036 |
|  | 2-Hydroxypropyl 2-isopropyl-5-methylcyclohexyl carbonate | 137.740 | 162.222 |
|  | 4-Hydroxy-3-polyprenylbenzoate | 98.546 | 224.353 |
|  | (+)-Myrtenyl formate | 20.914 | 23.599 |
|  | Hyperforin | 88.545 | 5.173 |
|  | Vanillin 3-(L-menthoxy)propane-1,2-diol acetal | 10.578 | 14.242 |
|  | Acoric acid | 72.129 | 100.951 |
|  | Menthyl ethylene glycol carbonate | 116.907 | 45.944 |
|  | Valdiate | 71.706 | 6.629 |
|  | (1S,2S,4R,8R)-p-Menthane-1,2,8,9-tetrol | 79.307 | 0.161 |
|  | 2,2,4,4, Tetramethyl-6-(1-oxopropyl)-1,3,5-cyclohexanetrione | 32.829 | 6.810 |
|  | Monomenthyl succinate | 160.498 | 170.561 |
|  | 3-[[5-Methyl-2-(1-methylethyl)cyclohexyl]oxy]-1,2-propanediol | 24.927 | 14.952 |
|  | 2,2,4,4-Tetramethyl-6-(1-oxobutyl)-1,3,5-cyclohexanetrione | 75.872 | 105.160 |
|  | L-Menthyl acetoacetate | 21.184 | 26.409 |
|  | (+)-Neomenthol | 32.227 | 24.202 |
|  | 7,11-Bisdeacetylvaltrate 7-(3-methylpentanoate) 11-(3-hydroxy-3-methylbutanoate) | 26.809 | 10.712 |
|  | 1-Hydroxypropan-2-yl 2-isopropyl-5-methylcyclohexyl carbonate | 200.849 | 219.340 |
|  | (3S,6E)-Nerolidol | 49.742 | 40.951 |
|  | Norecasantalic acid | 3.582 | 0.000 |
|  | 2-Polyprenyl-6-methoxyphenol | 66.490 | 28.303 |
|  | Genipin | 46.913 | 39.433 |
|  | 2,6-di-tert-butylbenzoquinone | 4.218 | 30.537 |
|  | Dihydrovaltrate | 49.415 | 58.994 |
|  | Piperochromenoic acid | 5.591 | 10.125 |
|  | (E)-4,8-Dimethyl-1,3,7-nonatriene | 32.609 | 33.774 |
|  | 4-Methyl-2-methylene-1-(1 methylethylidene)-cyclohexane | 11.188 | 15.990 |
| polyterpenoids | 2-decaprenyl-5-hydroxy-6-methoxy-3-methyl-1,4-benzoquinone | 2245.339 | 2724.462 |
| sesquiterpenoids | beta-Farnesene | 28540.255 | 72048.613 |
|  | Copaene | 13273.888 | 19300.862 |
|  | alpha-Curcumene | 6363.721 | 1803.078 |
|  | Preisocalamendiol | 3378.741 | 1190.174 |
|  | Rosifoliol | 23058.126 | 39729.481 |
|  | Acoragermacrone | 1658.759 | 1821.249 |
|  | Humuladienone | 7221.130 | 5266.958 |
|  | Auberganol | 4499.244 | 6481.115 |
|  | Calamusenone | 3606.753 | 1405.110 |
|  | Lubiminol | 141.283 | 217.750 |
|  | Calamendiol | 20.270 | 26.492 |
|  | Epishyobunone | 12643.299 | 12669.611 |
|  | Lacinilene C | 1226.591 | 412.604 |
|  | Zedoarol | 1130.933 | 501.964 |
|  | Curdione | 109.686 | 57.208 |
|  | Methyl (3b,11x)-3-Hydroxy-8-oxo-6-eremophilen-12-oate | 0.698 | 170.608 |
|  | (3S,4S,6R,7S)-1,10-Bisaboladiene-3,4-diol | 418.332 | 188.509 |
|  | 4,7(11)-Guaiadien-8-one | 1781.334 | 521.966 |
|  | 6alpha-Carissanol | 24.505 | 31.799 |
|  | Germacrenone | 66.855 | 0.556 |
|  | Procurcumadiol | 86.251 | 56.877 |
|  | 13-Nor-6-Eremophilene-8,11-dione | 102.915 | 983.794 |
|  | beta-Ionol | 323.801 | 1971.908 |
|  | Zedoarondiol | 1537.819 | 1692.057 |
|  | (3beta,6beta)-Furanoeremophilane-3,6-diol 6-acetate | 402.171 | 167.577 |
|  | Curcolone | 72.993 | 13.494 |
|  | Armillaritin | 18.565 | 18.408 |
|  | 3'-Hydroxy-HT2 toxin | 724.229 | 145.630 |
|  | Tanacetol A | 3.191 | 3.117 |
|  | 13-Hydroxy-5'-O-methylmelledonal | 704.228 | 176.852 |
|  | 5'-O-Methylmelledonal | 185.949 | 99.811 |
|  | Epioxylubimin | 579.077 | 626.146 |
|  | Eremopetasidione | 28.546 | 318.632 |
|  | delta-Methylionone | 416.709 | 71.298 |
|  | 4,5-Dihydrovomifoliol | 71.252 | 49.895 |
|  | 5beta-1,3,7(11)-Eudesmatrien-8-one | 204.882 | 295.231 |
|  | Petasitin | 163.286 | 6.954 |
|  | Nigellic acid | 6.509 | 84.266 |
|  | 3,11,12-Trihydroxy-1(10)-spirovetiven-2-one | 96.388 | 198.317 |
|  | (1a,5b,6a)-7-Protoilludene-1,5,6,14-tetrol 14-(2,4-dihydroxy-6-methylbenzoic acid) | 6.604 | 0.040 |
|  | Deoxynivalenol 3-glucoside | 25.221 | 82.235 |
|  | 3'-Hydroxy-T2-triol | 1.650 | 1.329 |
|  | 2,3-Dihydroabscisic alcohol | 2835.410 | 4362.915 |
|  | Tocopheronic acid | 9.699 | 16.928 |
|  | (ent-2b,4S,9a)-2,4,9-Trihydroxy-10(14)-oplopen-3-one 2-(2-methylbutanoate) 9-(3-methyl-2E-pentenoate) | 76.872 | 31.834 |
|  | Nerolidol | 72.204 | 41.978 |
|  | 1,6,9-Farnesatriene-3,11-diol | 140.056 | 126.050 |
|  | (4E,9a)-9-(3-Methyl-2E-pentenoyloxy)-4,10(14)-oplopadien-3-one | 122.172 | 15.116 |
|  | Armillarin | 4834.174 | 4808.185 |
|  | Acetylvalerenolic acid | 10.967 | 13.110 |
|  | Melledonal A | 7.861 | 17.375 |
|  | (7b,10a)-3-Hydroxy-1,3,5-cadinatrien-9-one | 79.424 | 84.198 |
|  | Pisumic acid | 25.548 | 25.804 |
|  | beta-Ionyl acetate | 27.704 | 1.908 |
|  | 4-O-Methylmelleolide | 112.347 | 38.975 |
|  | Anhydroretinol | 188.723 | 93.447 |
|  | 7(14)-Bisabolene-2,3,10,11-tetrol | 42.860 | 251.409 |
|  | Artesunate | 285.026 | 127.716 |
|  | Cinncassiol E | 0.979 | 0.332 |
|  | Melleolide | 72.718 | 14.999 |
|  | Gossyvertin | 82.901 | 44.054 |
|  | Toxin T2 tetrol | 53.634 | 1.936 |
|  | Abscisic alcohol | 6.431 | 36.295 |
|  | 8-Propanoylneosolaniol | 85.984 | 2.128 |
|  | (1(10)E,4a,5E)-1(10),5-Germacradiene-12-acetoxy-4,11-diol | 107.819 | 7.167 |
|  | Diacetoxyscirpenol | 34.381 | 6.197 |
|  | 8-Pentanoylneosolaniol | 148.657 | 49.499 |
|  | Tanacetol B | 6.932 | 4.650 |
|  | Eremopetasin sulfoxide | 16.035 | 46.540 |
|  | Dihydroartemisinin (DHA) | 17.833 | 21.687 |
|  | 3,7,8,15-Scirpenetetrol | 27.683 | 37.774 |
|  | 3'-Hydroxy-T2 toxin | 34.591 | 9.210 |
|  | Armillaripin | 13.527 | 18.669 |
|  | T-2 toxin | 37.278 | 15.841 |
|  | Dehydrovomifoliol | 19.897 | 26.544 |
|  | S-Furanopetasitin | 4197.521 | 2640.362 |
|  | S-Japonin | 23.410 | 21.877 |
|  | 7,8-Dehydro-3,4-dihydro-beta-ionol | 52.301 | 68.223 |
|  | 4,8-Diacetyl-T2-tetrol | 8.216 | 2.648 |
|  | Hernandulcin | 181.074 | 220.978 |
|  | Deterrol stearate | 163.478 | 169.242 |
|  | Melleolide M | 41.849 | 27.560 |
|  | Armillaric acid | 43.855 | 0 |
|  | 15-Acetoxyscirpene-3,4-diol 4-O-a-D-glucopyranoside | 5.870 | 0.708 |
|  | Melleolide D | 128.847 | 96.736 |
|  | 6-O-Methylarmillaridin | 126.503 | 187.499 |
|  | 25-Cinnamoyl-vulgaroside | 342.723 | 248.583 |
| triterpenoids | Ganoderic acid U | 7.299 | 12.340 |
|  | (24E)-3alpha,15alpha-Diacetoxy-23-oxo-7,9(11),24-lanostatrien-26-oic acid | 62.956 | 13.017 |
|  | Limonin | 70.448 | 76.146 |
|  | Lucidenic acid G | 28.289 | 64.525 |
|  | Ganoderenic acid E | 628.461 | 198.804 |
|  | Lucidenic acid H | 25.157 | 44.565 |
|  | Tsugarioside B | 695.845 | 778.703 |
|  | Ganoderic acid theta | 71.183 | 37.026 |
|  | Manglupenone | 212.909 | 229.681 |
|  | Majoroside F2 | 37.595 | 24.499 |
|  | Zapoterin | 312.470 | 330.709 |
|  | Lucidenic acid D2 | 3.704 | 16.779 |
|  | 20-Hydroxy-3,7,11,15,23-pentaoxolanost-8-en-26-oic acid | 49.548 | 30.099 |
|  | 3,7,11,15,23-Pentaoxolanost-8-en-26-oic acid | 4.432 | 27.454 |
|  | Phytolaccinic acid | 20.856 | 21.624 |
|  | Quinquenoside F1 | 12.592 | 8.953 |
|  | 4alpha-hydroxymethyl-4beta-methyl-5alpha-cholesta-8-en-3beta-ol | 11.862 | 12.226 |
|  | (3b,16a,21b,22a)-12-Oleanene-3,16,21,23,28-pentol-22-angeloyloxy-23-al | 88.367 | 108.961 |

The relative quantitative results (no units) were calculated based on the peak areas; and the values shown are the averages of the values obtained for 6 replicates (n = 6). GM, #X1, evergreen mutant; YWt, #3, yellowish-brown needles in winter.

**Supplementary Table 3.** Differentially synthesized metabolites (DSMs) involved in terpenoid-related pathways.

| **Subclass** | **Metabolite** | **GM** | **YWt** |
| --- | --- | --- | --- |
| diterpenoids | Isopimaric acid | 0 | 82.973 |
|  | Cavipetin A | 0 | 66.822 |
|  | Gibberellin A39 | 5.407 | 0 |
|  | 3,3'-Bis(4''-hydroxyanigorufone) | 4.174 | 0 |
|  | Tricrocin | 287.029 | 4.507 |
|  | Carnosic acid | 3079.327 | 87.702 |
|  | 12-Hydroxy-11-methoxy-8,11,13-abietatrien-20-oic acid | 191.190 | 10.442 |
|  | 11beta-Hydroxygibberellin A7 | 22.269 | 2.124 |
|  | Siderol | 152.726 | 17.569 |
|  | ent-16b,19-Kauranediol 19-acetate | 50.025 | 7.782 |
| monoterpenoids | Norecasantalic acid | 3.582 | 0 |
|  | (1S,2S,4R,8R)-p-Menthane-1,2,8,9-tetrol | 79.307 | 0.161 |
|  | Hyperforin | 88.545 | 5.173 |
|  | Valdiate | 71.706 | 6.629 |
|  | 2,2,4,4, Tetramethyl-6-(1-oxopropyl)-1,3,5-cyclohexanetrione | 32.829 | 6.810 |
|  | 2,6-di-tert-butylbenzoquinone | 4.218 | 30.537 |
| sesquiterpenoids | Armillaric acid | 43.855 | 0 |
|  | Germacrenone | 66.855 | 0.556 |
|  | (1a,5b,6a)-7-Protoilludene-1,5,6,14-tetrol 14-(2,4-dihydroxy-6-methylbenzoic acid) | 6.604 | 0.040 |
|  | 15-Acetoxyscirpene-3,4-diol 4-O-a-D-glucopyranoside | 5.870 | 0.708 |
|  | Methyl (3b,11x)-3-Hydroxy-8-oxo-6-eremophilen-12-oate | 0.698 | 170.608 |
|  | 8-Propanoylneosolaniol | 85.984 | 2.128 |
|  | Toxin T2 tetrol | 53.634 | 1.936 |
|  | Petasitin | 163.286 | 6.954 |
|  | beta-Ionyl acetate | 27.704 | 1.908 |
|  | (1(10)E,4a,5E)-1(10),5-Germacradiene-12-acetoxy-4,11-diol | 107.819 | 7.167 |
|  | Nigellic acid | 6.509 | 84.266 |
|  | Eremopetasidione | 28.546 | 318.632 |
|  | 13-Nor-6-eremophilene-8,11-dione | 102.915 | 983.794 |
|  | (4E,9a)-9-(3-Methyl-2E-pentenoyloxy)-4,10(14)-oplopadien-3-one | 122.172 | 15.116 |
|  | beta-Ionol | 323.801 | 1971.908 |

The relative quantitative results (no units) were calculated based on the peak areas; the values shown are the averages of the values obtained for 6 replicates (n = 6). GM, #X1, evergreen mutant; YWt, #3, yellowish-brown needles in winter.

Supplementary Table S4

In Supplementary Table S4.

**Supplementary Table 5.** Summary of the sequence quality analysis of 6 RNA libraries of *C. fortunei*.

| **Sample** | **Raw reads (M)** | **Raw bases (G)** | **Clean reads (M)** | **Clean bases (G)** | **Valid bases (%)** | **Q30 (%)** | **GC (%)** |
| --- | --- | --- | --- | --- | --- | --- | --- |
| GM_1 | 55.16 | 8.27 | 54.80 | 7.86 | 95.01 | 95.57 | 44.25 |
| GM_2 | 49.87 | 7.48 | 49.55 | 7.15 | 95.54 | 95.72 | 44.27 |
| GM_3 | 49.97 | 7.50 | 49.65 | 7.09 | 94.64 | 95.69 | 44.29 |
| YWt_1 | 50.88 | 7.63 | 50.53 | 7.23 | 94.79 | 95.60 | 44.19 |
| YWt_2 | 49.66 | 7.45 | 49.30 | 7.02 | 94.22 | 95.81 | 44.33 |
| YWt_3 | 49.25 | 7.39 | 48.92 | 6.99 | 94.65 | 96.02 | 44.19 |

GM, #X1, evergreen mutant; YWt, #3, yellowish-brown needles in winter. GM_1, GM_2 and GM_3 represent 3 repetitions.

Supplementary Table S6

In Supplementary Table S6.

Supplementary Table S7

In Supplementary Table S7.

**Supplementary Table 8.** Data annotation of the transcriptome.

| **Annotation database** | **Annotated number** | **300 bp ≤ length < 1000 bp** | **Length ≥ 1000 bp** |
| --- | --- | --- | --- |
| NR | 27,144 (48.61%) | 8193 (14.67%) | 18,951 (33.94%) |
| SwissProt | 20,620 (36.93%) | 5355 (9.59%) | 15,265 (27.34%) |
| KEGG | 8996 (16.11%) | 2075 (3.72%) | 6921 (12.39%) |
| KOG | 15,751 (28.21%) | 4358 (7.80%) | 11,393 (20.40%) |
| eggNOG | 24,795 (44.40%) | 6808 (12.19%) | 17,987 (32.21%) |
| GO | 17,811 (31.90%) | 4264 (7.64%) | 13,547 (24.26%) |
| Pfam | 19,817 (35.49%) | 4187 (7.50%) | 15,630 (27.99%) |

**Supplementary Table 9.** Summary of the sRNA sequencing quality of *C. fortunei*.

|  | **Sample** | **GM_1** | **GM_2** | **GM_3** | **YWt_1** | **YWt_2** | **YWt_3** |
| --- | --- | --- | --- | --- | --- | --- | --- |
| annotation | raw reads (M) | 39.99 | 44.55 | 48.73 | 37.43 | 42.29 | 41.6 |
|  | reads trimmed length (M) | 21.05 | 23.15 | 22.4 | 24.18 | 27.06 | 24.98 |
|  | reads trimmed Q20 (M) | 21.01 | 23.11 | 22.36 | 24.13 | 27.00 | 24.93 |
|  | reads trimmed N (M) | 21.00 | 23.10 | 22.35 | 24.12 | 26.99 | 24.92 |
|  | clean reads (M) | 21.00 | 23.10 | 22.35 | 24.12 | 26.99 | 24.92 |
| aligned transcriptome | aligned reads | 17,999,995 | 19,693,712 | 19,350,705 | 20,203,484 | 22,562,518 | 20,867,055 |
|  | aligned rate (%) | 85.72 | 85.25 | 86.58 | 83.75 | 83.59 | 83.73 |
| aligned Rfam | rRNA | 7457 | 5936 | 3892 | 7624 | 8995 | 8771 |
|  | tRNA | 1444 | 1587 | 1364 | 1474 | 1711 | 2183 |
|  | snRNA | 8711 | 13,306 | 9889 | 12,144 | 13,847 | 10,679 |
|  | *cis*-reg | 9973 | 13,178 | 12,355 | 16,229 | 19,162 | 16,292 |
|  | other Rfam RNA | 74,959 | 76,285 | 81,008 | 51,746 | 59,300 | 57,279 |
| aligned gene | aligned reads | 457,103 | 444,263 | 313,734 | 1,084,236 | 1,471,807 | 734,132 |
|  | aligned rate (%) | 2.18 | 1.92 | 1.40 | 3.98 | 4.80 | 2.75 |
| aligned repeat | aligned reads | 1,108,089 | 1,272,524 | 1,301,555 | 1,285,138 | 1,494,439 | 1,391,521 |
|  | aligned rate (%) | 5.28 | 5.51 | 5.82 | 5.33 | 5.54 | 5.58 |
| aligned known miRNA | aligned reads | 398,256 | 354305 | 390,803 | 530493 | 584,470 | 534,549 |
|  | aligned rate (%) | 1.9 | 1.53 | 1.75 | 2.2 | 2.17 | 2.15 |
| nonannotated | aligned reads | 398,256 | 20,918,884 | 20,235,505 | 21,464,249 | 23,792,535 | 22,132,657 |
|  | aligned rate (%) | 90.16 | 90.56 | 90.54 | 88.97 | 88.14 | 88.81 |

GM, #X1, evergreen mutant; YWt, #3, yellowish-brown needles in winter. GM_1, GM_2 and GM_3 represent 3 repetitions.

**Supplementary Table 10.** Summary of the degradome sequencing quality of *C. fortunei*.

| **Sample** | **GM (number)** | **GM (%)** | **YWt (number)** | **YWt (%)** |
| --- | --- | --- | --- | --- |
| raw reads | 29,051,452 | / | 55,549,521 | / |
| unique raw reads | 9,473,983 | / | 31,301,265 | / |
| reads < 15 nt after removing 3’ adaptor | 250,717 | 0.86 | 607,291 | 1.09 |
| mappable reads | 28,800,735 | 99.14 | 54,942,230 | 98.91 |
| unique reads < 15 nt after removing 3’ adaptor | 84,181 | 0.89 | 238,325 | 0.76 |
| unique mappable reads | 9,389,802 | 99.11 | 31,062,940 | 99.24 |
| mapped reads | 24,166,407 | 83.18 | 18,651,914 | 33.58 |
| unique mapped reads | 7,223,211 | 76.24 | 5,795,795 | 18.52 |
| number of input transcripts | 55,839 | / | 55,839 | / |
| number of covered transcripts | 42,337 | 75.82 | 41,195 | 73.77 |

GM, #X1, evergreen mutant; YWt, #3, yellowish-brown needles in winter.

**Supplementary Table 11.** Statistics for each category of the degradome.

| **Category** | **GM** | **YWt** |
| --- | --- | --- |
| 0 | 206 | 203 |
| 1 | 24 | 36 |
| 2 | 2159 | 1780 |
| 3 | 968 | 763 |
| 4 | 2676 | 2305 |
| NA | 2313 | 3259 |
| Total | 8346 | 8346 |

GM, #X1, evergreen mutant; YWt, #3, yellowish-brown needles in winter.

**Supplementary Table 12.** MiRNAs that identify target genes as transcription factors through the degradome.

| **MiRNA** | | | **Transcript** | | | |
| --- | --- | --- | --- | --- | --- | --- |
| **MiRNA** | **GM** | **YWt** | **Transcript** | **TF** | **GM** | **YWt** |
| aau-miR172 | 1.656 | 0.264 | TRINITY_DN21816_c0_g1_i11_1 | *AP2* | 0.350 | 0.633 |
|  |  |  | TRINITY_DN23681_c0_g1_i3_3 |  | 13.658 | 18.763 |
| novel73_star | 0.150 | 0.104 | TRINITY_DN18658_c0_g1_i1_2 |  | 0.708 | 0.718 |
| aly-miR172e-3p | 1.817 | 0 | TRINITY_DN23681_c0_g1_i3_3 |  | 13.658 | 18.763 |
| ata-miR172a-3p | 0.146 | 0.233 | TRINITY_DN20416_c0_g1_i1_3 |  | 3.060 | 2.925 |
|  |  |  | TRINITY_DN21816_c0_g1_i11_1 |  | 0.350 | 0.633 |
|  |  |  | TRINITY_DN23681_c0_g1_i3_3 |  | 13.658 | 18.763 |
| cpa-miR172a | 0.016 | 0 | TRINITY_DN20416_c0_g1_i1_3 |  | 3.060 | 2.925 |
|  |  |  | TRINITY_DN23681_c0_g1_i3_3 |  | 13.658 | 18.763 |
| csi-miR172a-3p | 0.029 | 0.067 | TRINITY_DN23681_c0_g1_i3_3 |  | 13.658 | 18.763 |
| novel11_star | 11.399 | 14.107 | TRINITY_DN20416_c0_g1_i1_3 |  | 3.060 | 2.925 |
| sbi-miR172f | 0 | 0.014 | TRINITY_DN21742_c0_g2_i2_1 |  | 0.532 | 0.955 |
| aly-miR858-5p | 0.776 | 1.396 | TRINITY_DN17020_c0_g1_i2_3 | *MYB* | 1.455 | 1.074 |
|  |  |  | TRINITY_DN17283_c0_g1_i3_3 |  | 12.776 | 17.094 |
|  |  |  | TRINITY_DN19328_c0_g1_i4_2 |  | 14.704 | 10.189 |
|  |  |  | TRINITY_DN23694_c0_g2_i3_2 |  | 12.596 | 12.454 |
|  |  |  | TRINITY_DN25624_c0_g1_i3_1 |  | 16.399 | 14.130 |
| ath-miR858b | 31.025 | 17.061 | TRINITY_DN16647_c0_g1_i2_1 |  | 6.040 | 6.376 |
|  |  |  | TRINITY_DN16820_c0_g1_i2_3 |  | 10.263 | 8.888 |
|  |  |  | TRINITY_DN17020_c0_g1_i2_3 |  | 1.455 | 1.074 |
|  |  |  | TRINITY_DN19328_c0_g1_i4_2 |  | 14.704 | 10.189 |
|  |  |  | TRINITY_DN25624_c0_g1_i3_1 |  | 16.399 | 14.130 |
|  |  |  | TRINITY_DN27816_c0_g2_i1_3 |  | 1.409 | 1.656 |
| cme-miR159b | 0.221 | 0.039 | TRINITY_DN2913_c0_g1_i1_3 |  | 3.480 | 3.672 |
| cme-miR319c | 0.485 | 0.037 | TRINITY_DN17554_c0_g1_i1_1 |  | 3.310 | 3.167 |
|  |  |  | TRINITY_DN2913_c0_g1_i1_3 |  | 3.480 | 3.672 |
| cme-miR828 | 3.384 | 2.280 | TRINITY_DN16647_c0_g1_i2_1 |  | 6.040 | 6.376 |
|  |  |  | TRINITY_DN16820_c0_g1_i2_3 |  | 10.263 | 8.888 |
|  |  |  | TRINITY_DN18260_c0_g1_i2_1 |  | 1.313 | 1.186 |
|  |  |  | TRINITY_DN19041_c0_g1_i1_3 |  | 14.837 | 15.418 |
|  |  |  | TRINITY_DN21383_c0_g1_i5_1 |  | 14.182 | 10.227 |
|  |  |  | TRINITY_DN24199_c4_g1_i1_3 |  | 4.385 | 2.772 |
|  |  |  | TRINITY_DN26478_c0_g1_i3_2 |  | 3.476 | 1.853 |
|  |  |  | TRINITY_DN26478_c0_g3_i2_2 |  | 0.610 | 0.926 |
| csi-miR166i-3p | 0.044 | 0.077 | TRINITY_DN24697_c0_g1_i2_1 |  | 11.495 | 10.644 |
| csi-miR858-3p | 0.102 | 0.040 | TRINITY_DN16884_c0_g1_i2_2 |  | 6.826 | 7.097 |
|  |  |  | TRINITY_DN20151_c0_g1_i4_3 |  | 1.729 | 1.279 |
|  |  |  | TRINITY_DN21383_c0_g1_i5_1 |  | 14.182 | 10.227 |
| gma-miR319p | 0.016 | 0.012 | TRINITY_DN17554_c0_g1_i1_1 |  | 3.310 | 3.167 |
|  |  |  | TRINITY_DN22354_c0_g1_i4_3 |  | 9.785 | 10.494 |
|  |  |  | TRINITY_DN2913_c0_g1_i1_3 |  | 3.480 | 3.672 |
| gma-miR319q | 0 | 0.027 | TRINITY_DN22354_c0_g1_i4_3 |  | 9.785 | 10.494 |
|  |  |  | TRINITY_DN2913_c0_g1_i1_3 |  | 3.480 | 3.672 |
| gma-miR6300 | 12.193 | 8.593 | TRINITY_DN16647_c0_g1_i2_1 |  | 6.040 | 6.376 |
| lus-miR828a | 0.030 | 0.064 | TRINITY_DN12223_c0_g1_i1_1 |  | 0.908 | 1.698 |
|  |  |  | TRINITY_DN23365_c0_g2_i5_3 |  | 0.450 | 4.524 |
|  |  |  | TRINITY_DN25624_c0_g1_i3_1 |  | 16.399 | 14.130 |
|  |  |  | TRINITY_DN4888_c0_g1_i1_1 |  | 0.230 | 0.349 |
| mdm-miR535d | 0.150 | 0.225 | TRINITY_DN19041_c0_g1_i1_3 |  | 14.837 | 15.418 |
| mdm-miR858 | 0.088 | 0.290 | TRINITY_DN16820_c0_g1_i2_3 |  | 10.263 | 8.888 |
|  |  |  | TRINITY_DN22925_c0_g2_i2_3 |  | 7.682 | 11.595 |
|  |  |  | TRINITY_DN23694_c0_g2_i3_2 |  | 12.596 | 12.454 |
|  |  |  | TRINITY_DN23694_c0_g4_i1_2 |  | 15.200 | 17.877 |
|  |  |  | TRINITY_DN24171_c0_g1_i1_1 |  | 17.230 | 26.551 |
|  |  |  | TRINITY_DN25624_c0_g1_i3_1 |  | 16.399 | 14.130 |
|  |  |  | TRINITY_DN27816_c0_g2_i1_3 |  | 1.409 | 1.656 |
| mes-miR535d | 0.032 | 0.000 | TRINITY_DN19041_c0_g1_i1_3 |  | 14.837 | 15.418 |
| novel137_mature | 3.255 | 1.224 | TRINITY_DN17163_c0_g1_i1_1 |  | 7.682 | 7.546 |
| novel49_mature | 1.523 | 2.173 | TRINITY_DN22487_c0_g1_i2_3 |  | 11.422 | 12.150 |
| novel5_mature | 173.370 | 223.937 | TRINITY_DN14848_c0_g1_i1_1 |  | 5.129 | 4.143 |
| novel93_mature | 0.507 | 0.188 | TRINITY_DN15044_c0_g2_i1_3 |  | 18.507 | 23.275 |
| osa-miR159d | 0 | 0.494 | TRINITY_DN17554_c0_g1_i1_1 |  | 3.310 | 3.167 |
|  |  |  | TRINITY_DN2913_c0_g1_i1_3 |  | 3.480 | 3.672 |
| pab-miR159a | 84.617 | 84.705 | TRINITY_DN17554_c0_g1_i1_1 |  | 3.310 | 3.167 |
|  |  |  | TRINITY_DN22354_c0_g1_i4_3 |  | 9.785 | 10.494 |
|  |  |  | TRINITY_DN2913_c0_g1_i1_3 |  | 3.480 | 3.672 |
| pab-miR482l | 0.074 | 0.068 | TRINITY_DN23694_c0_g4_i1_2 |  | 26.124 | 29.979 |
| pab-miR858a | 0.239 | 0.320 | TRINITY_DN23694_c0_g1_i3_2 |  | 18.507 | 23.275 |
|  |  |  | TRINITY_DN31812_c0_g1_i1_3 |  | 4.084 | 5.226 |
| pde-miR159 | 1045.866 | 1037.188 | TRINITY_DN22354_c0_g1_i4_3 |  | 9.785 | 10.494 |
| ptc-miR166p | 0.383 | 0.400 | TRINITY_DN24697_c0_g1_i2_1 |  | 11.495 | 10.644 |
| zma-miR159b-5p | 0.014 | 0.067 | TRINITY_DN22067_c0_g1_i2_3 |  | 19.572 | 20.282 |
| zma-miR159e-3p | 2.573 | 1.040 | TRINITY_DN22354_c0_g1_i4_3 |  | 9.785 | 10.494 |
| aof-miR396a | 283.248 | 366.921 | TRINITY_DN17071_c0_g1_i3_3 | *WRKY* | 26.735 | 27.323 |
| ata-miR166d-5p | 1.318 | 1.749 | TRINITY_DN17071_c0_g1_i3_3 |  | 26.735 | 27.323 |

*AP2*, apetala2; GM, #X1, evergreen mutant; *MYB*, v-myb avian myeloblastosis viral oncogene homolog; TF, transcription factor; YWt, #3, yellowish-brown needles in winter.

**Supplementary Table 13.** Metabolites involved in flavonoid biosynthesis.

| **Metabolite** | **GM** | **YWt** |
| --- | --- | --- |
| (+)-Catechin | 20.70 ± 5.75 | 230.26 ± 24.81 |
| afzelechin | 0.94 ± 0.93 | 0 |
| epicatechin | 6258.01 ± 866.39 | 74389.60 ± 13093.70 |
| homoeriodictyol | 28.07 ± 3.90 | 0 |
| luteoforol | 0 | 69.38 ± 32.59 |
| pentahydroxyflavanone | 0 | 5.50 ± 3.24 |
| pinocembrin | 37.01 ± 3.04 | 0 |

The relative quantitative results (no units) were calculated based on the peak areas; the values shown are the averages of the values obtained for 6 replicates (n = 6). GM, #X1, evergreen mutant; YWt, #3, yellowish-brown needles in winter.
